# Selective Inhibition of IL-6 Trans-Signaling Has No Beneficial Effect on the Posttraumatic Cytokine Release after Multiple Trauma in Mice

**DOI:** 10.3390/life11111252

**Published:** 2021-11-17

**Authors:** Jil-Madeline Homeier, Katrin Bundkirchen, Marcel Winkelmann, Tilman Graulich, Borna Relja, Claudia Neunaber, Christian Macke

**Affiliations:** 1 Trauma Department and Department of Anesthesiology and Intensive Care, Hannover Medical School, 30625 Hannover, Germany; Homeier.Jil-Madeline@mh-hannover.de; 2 Trauma Department, Hannover Medical School, 30625 Hannover, Germany; Bundkirchen.Katrin@mh-hannover.de (K.B.); Winkelmann.Marcel@mh-hannover.de (M.W.); Graulich.Tilman@mh-hannover.de (T.G.); Neunaber.Claudia@mh-hannover.de (C.N.); 3 Experimental Radiology, Department of Radiology and Nuclear Medicine, Otto von Guericke University, 39120 Magdeburg, Germany; Borna.Relja@med.ovgu.de

**Keywords:** multiple trauma, MODS, sgp130Fc, trans-signaling, inflammatory cytokines

## Abstract

While improvements in pre-hospital and in-hospital care allow more multiple trauma patients to advance to intensive care, the incidence of posttraumatic multiple organ dysfunction syndrome (MODS) is on the rise. Herein, the influence of a selective IL-6 trans-signaling inhibition on posttraumatic cytokine levels was investigated as an approach to prevent MODS caused by a dysbalanced posttraumatic immune reaction. Therefore, the artificial IL-6 trans-signaling inhibitor sgp130Fc was deployed in a murine multiple trauma model (femoral fracture plus bilateral chest trauma). The traumatized mice were treated with sgp130Fc (FP) and compared to untreated mice (WT) and IL-6 receptor knockout mice (RKO), which received the same traumas. The overall trauma mortality was 4.4%. Microscopic pulmonary changes were apparent after multiple trauma and after isolated bilateral chest trauma. Elevated IL-6, MCP-3 and RANTES plasma levels were measured after trauma, indicating a successful induction of a systemic inflammatory reaction. Significantly reduced IL-6 and RANTES plasma levels were visible in RKO compared to WT. Only a little effect was visible in FP compared to WT. Comparable cytokine levels in WT and FP indicate neither a protective nor an adverse effect of sgp130Fc on the cytokine release after femoral fracture and bilateral chest trauma.

## 1. Introduction

Injuries cause 16% of the global burden of diseases [1]. Despite constant improvements in pre-hospital and in-hospital trauma care, the mortality rate in severely injured patients who reach the hospital, even in high-income countries, still accounts for 15% [2]. As a result, trauma ranks first in causes for loss of potential life in Germany [2]. Whilst continuing enhancements in primary trauma care could be achieved by the application of refined treatment algorithms [3], the case fatality rate in patients, developing multiple organ dysfunction syndrome (MODS) in the secondary stage after trauma, remains considerably high [4,5,6,7].

In the current state of research, an imbalance in pro- and anti-inflammatory responses is presumed etiological for severe trauma complications described as early and late MODS [8,9,10]. Traumatic tissue damage and hemorrhage liberate endogenous mediators to the bloodstream that are physiologically located intracellularly and are known as damage-associated molecular patterns (DAMPs) or ‘alarmins’ [11,12]. Similar to pathogen-associated molecular patterns (PAMPs), DAMPs trigger proinflammatory pathways, explaining the phenomenon of a ‘sterile inflammation’ after trauma [12,13,14]. According to posttraumatic immune response theories, the exacerbation of innate proinflammatory immune reactions can result in systemic inflammatory response syndrome (SIRS), that facilitates early organ dysfunction [8,15]. In order to maintain homeostasis, the adaptive immune system responds with a compensatory systemic reaction, which can lead to late MODS [8]. A sustained proinflammatory reaction leads to persistent compensatory responses that can result in a state called severe systemic anti-inflammatory response syndrome (SARS) [8]. SARS again can facilitate late-organ dysfunction by predisposing them toward infectious complications [8]. 

The posttraumatic immune reaction is a delicate system, but mitigating the early proinflammatory reaction might hold opportunities to prevent early and late MODS. The signal molecule IL-6 acts as a pivotal player in the posttraumatic immune reaction [16]. Injuries such as chest trauma cause an increase in IL-6 and other systemic inflammatory cytokine levels by the activation of specific macrophages, e.g., hepatic Kupffer cells [17,18]. These Kupffer cells, in turn, secrete other cytokines such as MCP-1, which further contribute to systemic inflammation and organ damage [17]. Being the major inducer of hepatic acute phase proteins [19,20], IL-6 initiates the innate immunity and, by orchestrating its transition to the acquired immunity, also resolves it [21]. Systemic IL-6 levels positively relate to trauma severity just as to the severity of posttraumatic inflammation [16]. High concentrations are associated with a higher rate in MODS and mortality following trauma [16]. Therefore, influencing IL-6 signaling appears to be a promising approach to modulate the inflammatory processes leading to posttraumatic MODS.

However, IL-6 is described to generate both pro- and anti-inflammatory properties, mediating both impairing and valuable processes in the post injury organism [22,23], which underscores the complicacy of this approach. IL-6 signaling is known to operate via two main signaling pathways: In the ‘IL-6 classic signaling pathway’, IL-6 generates anti-inflammatory properties with potentially beneficial effects on trauma regeneration, bone metabolism and the prevention of infectious complications [23,24,25,26], whereas the ‘IL-6 trans-signaling pathway’, with a potentially compromising impact, is regarded to mediate proinflammatory IL-6 activities [23]. In classic signaling, IL-6 binds its α-receptor, the membrane-bound interleukin-6 receptor (IL-6R), which recruits a dimer of its β-receptor, the transmembrane signal transducer glycoprotein 130 (gp130), to mediate its effects [27,28]. IL-6R is selectively expressed in hepatocyte, microglia, particular leukocyte and particular epithelia cell surfaces only, limiting its mediated activities to a confined quantity of target cells [27,28,29]. In trans-signaling, IL-6 utilizes a soluble form of IL-6R (sIL-6R) that is present in the plasma to interact with the ubiquitously expressed gp130 [30]. Hence, further cell types, intrinsically unresponsive to IL-6 itself, can be activated by IL-6 trans-signaling. In addition, IL-6 trans-signaling is described as potentially resulting in a more extensive activation of intracellular cascades than IL-6 classic signaling, since it is independent from the disparity between expressed gp130 and IL-6R on the cell surface and since receptor internalization in IL-6 trans-signaling is a slower process [31]. sIL-6R is mainly formed by the limited proteolysis of membrane-bound IL-6Rs or rarely results from the translation of mRNA lacking transmembrane domain information, which is presumably caused by alternative splicing [32,33,34].

Under physiological conditions, IL-6 trans-signaling occurs temporarily and is locally limited [35]. Extended IL-6 trans-signaling is a self-sustaining process, maintaining chronic inflammatory diseases [35]. IL-6 trans-signaling was identified as crucial for the development and progression of chronic inflammatory diseases and inflammation-associated cancer [36]. Since a soluble variant of gp130 (sgp130), which features a sIL-6R-binding capacity, is available in the blood plasma, there is a natural inhibitor to limit IL-6 trans-signaling [37]. The construction and optimization of an artificial sgp130 variant, sgp130Fc, that can be utilized for a therapeutic IL-6 trans-singling blockade has been described before [37,38]. Sgp130Fc is a fusion protein that consists of two extracellular domains of gp130 and the Fc part of human immunoglobulin G1 (IgG1) [38]. The dimerized sgp130 variant displays a dose-dependent specific IL-6 trans-signaling blockade, with a demonstrated high efficiency in various models of inflammatory diseases [39]. The advantages of a selective IL-6 trans-signaling inhibition by sgp130Fc over a complete IL-6 blockade were previously demonstrated for fracture healing and survival in microbial sepsis models [25,40]. Therefore, we hypothesized that a selective IL-6 trans-signaling inhibition by the application of sgp130Fc may reduce the proinflammatory trauma response and, thus, also provide beneficial effects on the immune reaction after multiple trauma. 

The aim of the present study was to investigate the effects of a selective inhibition of IL-6 trans-signaling by the use of sgp130Fc on the posttraumatic cytokine levels in a well-established murine multiple trauma model, consisting of a femoral fracture and a blunt bilateral chest trauma. 

## 2. Materials and Methods

### 2.1. Animal Care

The present animal experiment was undertaken in accordance with § 8 (1) of the German Animal Welfare Law in the version published on 18 May 2006, as approved by the Veterinary Institute for Animal Welfare of the Lower Saxony State Office for Consumer Protection and Food Safety, Germany (Approval No. 33.12-42502-04-15/1839).

Two hundred and eight male C57BL/6J were purchased from Charles River Laboratories (Sulzfeld, Germany) and provided via the Central Laboratory Animal Facility at the Hannover Medical School. Two B6;SJL-Il6ra^tm1.1Drew^/J breeding pairs were purchased from Charles River Laboratories (Sulzfeld, Germany) and mated in the Central Laboratory Animal Facility at the Hannover Medical School to establish a breeding pair to obtain 88 male B6;SJL-Il6ra^tm1.1Drew^/J, which were included in the experiment. The mice utilized in this study were aged 12 weeks in mean. 

Adequate housing in social groups was provided until the direct beginning of the experiment. Animals were maintained under standardized conditions and a controlled environment at 20 ± 2 °C, relative humidity of 55–60% and a simulated day–night rhythm (14-h artificial light, 10-h dark). A commercial pellet diet (Altromin 1324 TPF maintenance diet for rats and mice, Altromin GmbH, Lage, Germany) and drinking water were provided *ad libitum*. Infrared warming lamps were installed to facilitate thermoregulation after the trauma-generating surgery. The animals’ body weights and activity levels were measured prior to the trauma and surveyed afterwards. The utilized activity scoring system was described before [41]. 

### 2.2. Group Distribution

**Figure 1:**
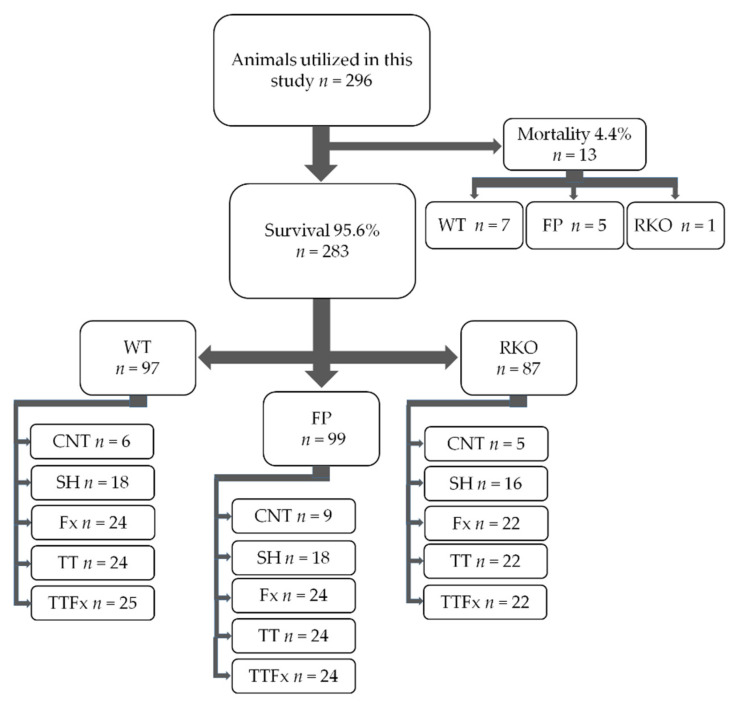
Study design and mortality. For this study, we compared wildtype mice with full IL-6-signaling capacities (WT) to wildtype mice with inhibited IL-6 trans-signaling (FP) and IL-6 receptor knockout mice (RKO). The mice were randomly allocated to the trauma, sham and control cohorts. CNT: healthy animals without trauma-generating surgery, SH: femur pin stabilization (Sham), Fx: femoral fracture, TT: bilateral chest trauma and TTFx: bilateral chest trauma plus femoral fracture. For detailed group distribution per time points, see Supplementary Table S1.

The study design is displayed in Figure 1. The group distribution and interventions are shown in the Supplementary Table S1. Regarding the IL-6 signaling capacity, three groups were differentiated in this study: (1) wildtype mice with unaffected IL-6 signaling capacities (WT), (2) wildtype mice treated with fusion protein sgp130Fc in order to inhibit IL-6 trans-signaling (FP) and (3) mice featuring a global IL-6 signaling blockade due to an IL-6 receptor knockout (RKO). The laboratory animals of each group were randomly allocated into five cohorts: control (CNT), sham (SH) and three trauma cohorts. The CNT cohort consisted of healthy animals that underwent no surgical intervention. Animals allocated to the SH cohort received a femur pin stabilization without a fracture. Animals in the femoral fracture trauma cohort (Fx) received a femur pin stabilization followed by a femoral fracture. Animals assigned to the thoracic trauma cohort (TT) received an isolated bilateral chest trauma. In the multiple trauma cohort (TTFx), a combined trauma consisting of a femur pin stabilization followed by a femoral fracture and a bilateral chest trauma was conducted. The mice treated with sgp130Fc received a subcutaneous injection of 0.5 mg/kg body weight right after induction of the aforementioned traumas. The fusion protein sgp130Fc was kindly provided by the Conaris Research Institute AG (Kiel, Germany). 

### 2.3. Anesthesia and Perioperative Analgesia

For the duration of the surgery, the laboratory animals’ respiratory, circulatory and central nervous system functions were monitored by observation throughout. Central nervous pain processing and perception were suppressed by the implementation of an inhalation anesthesia with isoflurane (Baxter Deutschland GmbH, Unterschleißheim, Germany), complemented by a subcutaneous metamizole injection (Ratiopharm GmbH, Ulm, Germany). For the induction of the anesthesia, each mouse was decoyed into a glass tube. With a flow rate of 0.6 L/min, oxygen fortified with 4% isoflurane was introduced into the tube. During the maintenance of the anesthesia, the isoflurane concentration was reduced to values between 1.5 and 2.5%, individually adjusted to the demand. For better access to the torso and extremities during the intervention, the volatile anesthetic was administered via a mask covering the animal’s nostrils and mouth. The loss of the righting reflex and the toe pinch reflex was utilized to indicate the attainment of the required anesthetic depth. Next, a subcutaneous injection of metamizole 200 mg/kg body weight was administered. After completion of the surgery, the supply of isoflurane was stopped, and the vital functions were continuously monitored until the recurrence of spontaneous activity. Perioperative analgesic therapy was perpetuated via an additive of 1 mg/kg of metamizole (Zentiva Pharma GmbH, Frankfurt am Main, Germany) per mL to the animal’s drinking water.

### 2.4. Control

The CNT cohort did not experience any surgery or trauma application. Sgp130Fc-treated CNT animals underwent a subcutaneous sgp130Fc injection under isoflurane anesthesia, as described before. 

### 2.5. Intramedullary Femur Pin

The cohorts SH, Fx and TTFx received a stabilization of the right femur. The procedure was described before [42]. In short, the distal femur was accessed via a longitudinal skin incision of 5–8 mm in length. Conserving the patellar ligament, a 24-G canula (Sterican^®^ B. Braun AG, Melsungen, Germany) was introduced via the intercondylar fossa and advanced to the medullary canal. The canula was cut on the bone level, and primary wound closure was conducted using single button sutures (Prolene 6-0, P-3, 45 cm, Ethicon, Norderstedt, Germany). 

### 2.6. Femoral Fracture

The cohorts Fx and TTFx afterwards received a fracture of the pin-stabilized right femur by the use of a blunt guillotine apparatus, as described previously [42,43]. In short, with an impact energy of 0.784 J, a 500-g guided weight dropped towards an impact disc, effectuating a standardized movement of a blunt guillotine blade downwards onto the animal’s limb. This resulted in a type A (AO classification) femoral fracture accompanied by a moderate soft-tissue trauma. 

### 2.7. Bilateral Chest Trauma

The cohorts TT and TTFx received a blunt chest trauma. The trauma was induced in a modified version of our original trauma model, as described previously [44]. The anesthetized animal was attached to a glass plate and positioned under a guillotine-like chest trauma apparatus. Through a guide tunnel, a weight of 300 g dropped under standardized conditions from a height of 50 cm towards a platform, resting on the animal’s thorax and impressing it by 5 mm in a sagittal direction with an impact energy of 1.47 Joule.

### 2.8. Activity Score

A previously described [45] scoring system, regarding spontaneous movements, reactions to their environment, food intake and vital signs, was applied to measure the level of activity in each animal before and after the intervention. Scores between six (very active) and one (moribund) were awarded before and periodically after the intervention. Animals repeatedly awarded a score of four or lower by two independent examiners were euthanized. 

### 2.9. Final Blood Sampling and Sacrifice

In order to examine the alterations in the cytokine expression capacity over time, the animals were sacrificed 6, 12 or 24 h after injury and sgp130Fc administration. During the second isoflurane anesthesia, the peritoneal cavity was accessed via a median laparotomy, extended by a subcostal transverse incision. One milliliter of blood was sampled via transdiaphragmatic cardiocentesis in a heparin (Rotexmedica GmbH Arzneimittelwerk, Trittau, Germany)-coated syringe and centrifuged at a speed of 7000 rpm for 5 min at 4 °C for plasma (P) collection. After sacrifice by cervical dislocation, the accuracy of the trauma was accessed, and the organ removal was commenced. 

### 2.10. Organ Removal, Bronchoalveolar Lavage (BAL) and Hepatic Rinsing

A sternotomy was performed to access the thoracic structures. The inferior vena cava and the liver veins were ligated (Marlin 5/0, USP, Catgut GmbH, Markneukirchen, Germany), and the thorax organs were removed *en bloc*. 

For BAL, both lungs were rinsed three times with 1-mL heparinized (Rotexmedica GmbH Arzneimittelwerk, Trittau, Germany) normal saline (NaCl 0.9% Fresenius Kabi AG, Bad Homburg vor der Höhe, Germany) per pass. The obtained bronchoalveolar lavage fluid (BALF) was transferred to a sterile 15-mL tube (Greiner Bio-One, Kremsmünster, Austria) and was stored on ice. The right lung and a portion of the median liver lobe were fixed in 4% formaldehyde for histological examination. The left lung, the spleen, the kidneys and the remaining portion of the median liver lobe were frozen in liquid nitrogen (The Linde Group, München, Germany) and stored at −80 °C until further use. The remaining liver was rinsed via the portal vein twice with 20-mL HBSS (Gibco/Life Technologies, Waltham, MA, USA) and afterwards with 20 mL of a collagenase IV (Biochrom GmbH, Berlin, Germany)-containing solution. Subsequently, the liver was removed, cut into 1 × 1-mm pieces and incubated in a collagenase IV (Biochrom GmbH, Berlin, Germany)-containing solution at 37 °C for 20 min for Kupffer cell (KC) isolation. 

### 2.11. BALF Processing and Isolation of Alveolar Macrophages

The obtained BALF was centrifuged at a speed of 2000 rpm for 15 min at 4 °C. One milliliter of supernatant was withdrawn and stored at −80 °C until the analysis was performed. Alveolar macrophages (AMs) were isolated as previously described [44]. In short, the cell pellet and fluid were resuspended, and the centrifugation was repeated. The obtained cell pellet was resuspended in Williams’ E solution (Biochrom GmbH, Berlin, Germany) containing 10% fetal bovine serum (Biochrom GmbH, Berlin, Germany) and antibiotics (50 U/mL penicillin, 50 μg/mL streptomycin and 5 μg/mL gentamycin; Biochrom GmbH, Berlin, Germany). Fifty thousand cells were plated in a 96-well plate. After one h, the culture medium including nonadherent cells was replaced with 1 μg/mL lipopolysaccharide (LPS) (Sigma-Aldrich, St. Louis, MO, USA) for stimulation. After a 24 h incubation period at 37 °C, 95% humidity and 5% CO_2_, the supernatants were removed and stored at −80 °C until the analysis was performed. In cases where the required yield of 50,000 cells could not be obtained by isolation, a correction factor was applied in further evaluations to establish comparability.

### 2.12. Isolation of Kupffer Cells

KCs were isolated as previously described with some modifications [44,46,47]. In brief, after the above-mentioned incubation in collagenase IV solution, the liver pieces were passed through a Sigma 50 mesh (Sigma-Aldrich, St. Louis, MO, USA), and 45-mL HBSS with 10% FBS was added. Centrifugation at a speed of 500 rpm for 3 min at 4 °C followed for hepatocyte removal. The supernatant was centrifuged at a speed of 2000 rpm for 15 min at 4 °C. The cell pellet (pellet I) was covered with 5-mL HBSS with 10% FBS. The supernatant was centrifuged at a speed of 2000 rpm for 15 min at 4 °C. The supernatant was removed, and the cell pellet (pellet II) was resuspended with 5-mL HBSS with 10% FBS. The resuspended pellets (I and II) were gently mixed and layered over 14% Histodenz (Sigma-Aldrich, Munich, Germany). After centrifugation at a speed of 3900 rpm for 45 min at 4 °C, KCs were isolated from the interphase and transferred to a 15-mL tube (Greiner Bio-One, Kremsmünster, Austria). Williams’ E solution was added to a volume of 15 mL, and the fluid was centrifuged at a speed of 2000 rpm for 15 min at 4 °C. The obtained cell pellet was resuspended in Williams’ E solution (Biochrom GmbH, Berlin, Germany) containing 10% fetal bovine serum (Biochrom GmbH, Berlin, Germany) and antibiotics (50 U/mL penicillin, 50 μg/mL streptomycin and 5 μg/mL gentamycin; Biochrom GmbH, Berlin, Germany). One hundred thousand cells were plated in a 96-well plate. After one h, the culture medium including nonadherent cells was replaced with 1 μg/mL LPS (Sigma-Aldrich, St. Louis, MO, USA) for stimulation. After a 24-h incubation period at 37 °C, 95% humidity and 5% CO_2_, the supernatants were removed and stored at −80 °C until the analysis was performed. In cases where the required yield of the cells could not be obtained by isolation, comparability for further evaluations was established by the application of a correction factor.

### 2.13. Immunoassay for Cytokine Measurement

A quantification of inflammatory cytokines was performed using ProcartaPlex Mix&Match Mouse 6-plex (Bender MedSystems GmbH, Vienna, Austria), detecting IL-6, MCP-3, MCP-1, MIP-1 β, RANTES and GM-CSF, according to the manufacturer’s instructions. A Bio-Plex^®^ 200 system (Bio-Rad Laboratories GmbH, Feldkirchen, Germany) suspension array system and Bio-Plex Manager™ software were utilized. The cytokines IL-6, MCP-3, MCP-1, MIP-1β, RANTES and GM-CSF are established as objects of investigation regarding the posttraumatic inflammatory response and related organ failure [16,44,48,49]. Cytokine levels were measured in the blood plasma and BALF. Cytokine productive capacities were determined for the AMs and KCs.

### 2.14. Histological Examination

Hepatic and pulmonary tissue samples were fixed with 4% buffered formaldehyde, dehydrated, embedded in paraffin and cut into 2 to 3 µm-thick sections using a Reichert Jung 2040 Microtome (Leica Microsystems, Wetzlar, Germany). The sections were transferred to microscopic slides and stained with hematoxylin and eosin. A histological evaluation of the pulmonary tissue samples was conducted using a light microscope. Each section was regarded first in a fortyfold magnification and second in four representative sectors in a hundredfold magnification. In each section, the degree of pulmonary tissue injury and inflammation was assessed, applying a scoring system that was previously described [50]. In short, the pulmonary damage was evaluated as ‘minimal’ (0 pts), ‘mild’ (1 pt), ‘moderate’ (2 pts), ‘severe’ (3 pts) or ‘maximal’ (4 pts) in the microscopic criteria alveolar congestion, hemorrhage and neutrophil invasion of blood vessels and alveoli, as well as alveolar wall-thickening and the formation of hyaline membranes. A total score between 0 and 16 pts resulted.

### 2.15. Statistical Analysis

The power analysis for the group size calculation was performed using the power analysis provided at http://biomath.info/power/ttest.htm, accessed on 15 October 2021, taking a power of 0.8 as the basis, referring to the parameters previously investigated [44]. Descriptive statistics and the nonparametric Kruskal–Wallis test, which does not assume a normal distribution of the residuals, followed by Dunn’s post hoc test for the correction of multiple comparisons, were performed utilizing IBM SPSS Statistics for Windows, Version 27.0 (IBM Corp. Armonk, NY, USA). The statistical significance was set at *p* ≤ 0.05. For data visualization, GraphPad PRISM for Windows, Version 9 (GraphPad Software, Inc., San Diego, CA, USA) was used.

## 3. Results

### 3.1. Survival, Activity and Histological Examination

In total, 296 animals were utilized for this study. Of those, 283 animals survived. Thirteen animals died during the procedure, which resulted in an overall mortality of 4.4% (Figure 1). Seven animals from the WT cohort (mortality: 6.7%), five from the FP cohort (mortality: 4.8%) and one from the RKO cohort (mortality: 1.1%) died. Itemized by the type of trauma, three animals of the TT group (cohort mortality: 4.1%) and ten of the TTFx group (cohort mortality 12.3%) died. Twelve animals died directly after the induction of the chest trauma. One animal was euthanized after demonstrating a low activity score during the postinterventional period. Death was caused by chest trauma consequences in every case. In the postmortem examination, cardiorrhexis, aortic rupture and hemothorax were identified as fatal injuries.

Except for one animal that was euthanized shortly after injury at an activity score of two, all the animals were awarded an activity score of four or higher in the postinjury period. One hour after the intervention, over 90% of the animals demonstrated themselves active or very active, with scores between five and six (data not shown). After 4 h, all animals had returned to their normal activity level prior to the trauma onset.

**Figure 2:**
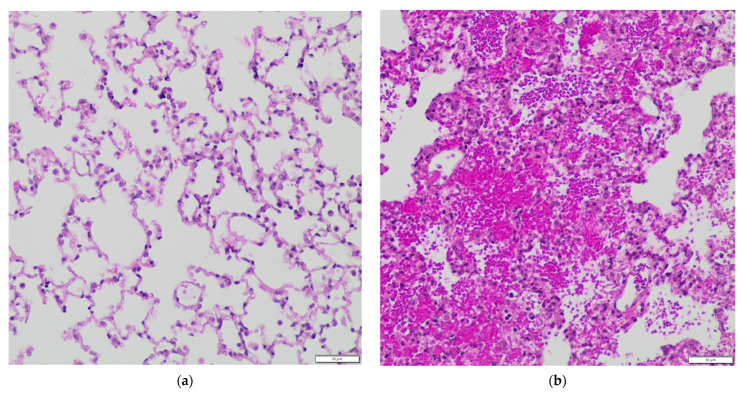

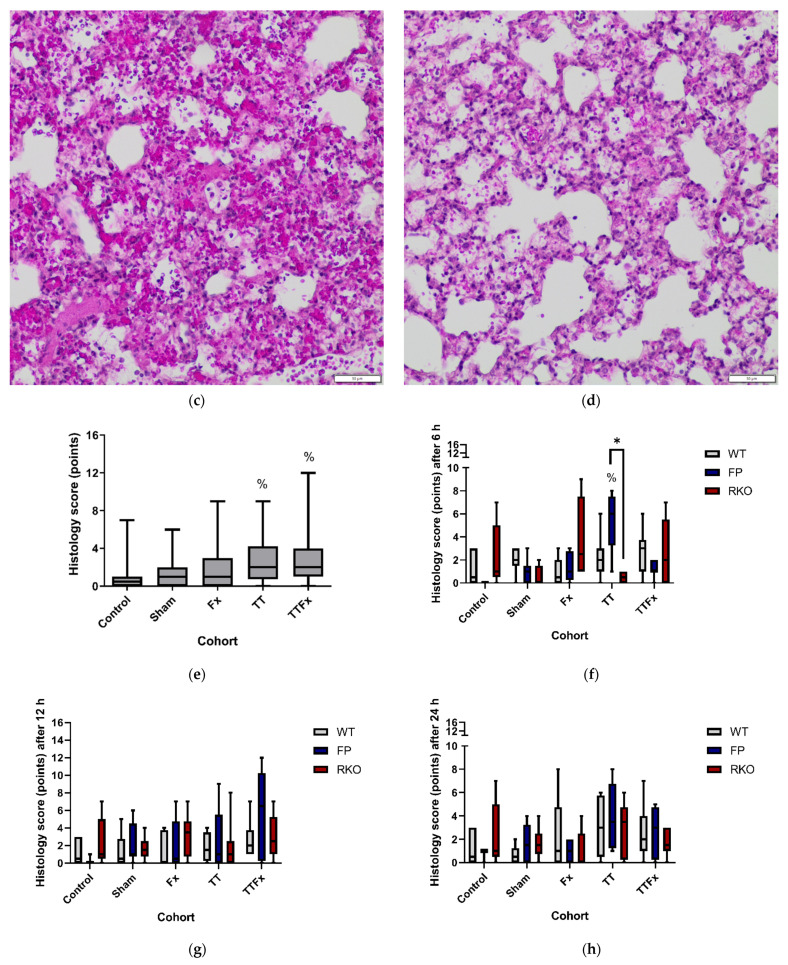
Posttraumatic alterations in the pulmonary histological examination. Multiple trauma and bilateral chest trauma induce significant organ damage to the lung. Sgp130Fc treatment does not influence the histological features after trauma. Control: healthy animals without trauma-generating surgery, Sham: femur pin stabilization, Fx: femoral fracture, TT: bilateral chest trauma and TTFx: bilateral chest trauma plus femoral fracture. *p* < 0.05 * vs. indicated or % vs. Control. (**a**) Microscopic images of the lung of CNT, healthy wildtype animal, no trauma-generating surgery. Histology score result is 0 pts. Hematoxylin–eosin stain, 100× magnification. (**b**) Microscopic images of the lung of TT, wildtype animal 6 h after bilateral chest trauma, treated with a single dose 0.5-mg/kg sgp130Fc. Histology score result is 8 pts. Hematoxylin–eosin stain, 100× magnification. (**c**) Microscopic images of the lung of TTFx, wildtype animal 12 h after bilateral chest trauma and femoral fracture, treated with a single dose 0.5-mg/kg sgp130Fc. Histology score result is 11 pts. Hematoxylin–eosin stain, 100× magnification. (**d**) Microscopic images of the lung of TT, wildtype animal 24 h after bilateral chest trauma, treated with a single dose 0.5-mg/kg sgp130Fc. Histology score result is 6 pts. Hematoxylin–eosin stain, 100× magnification. (**e**) Pulmonary damage scored by histological criteria. All 283 animals were included. Scale 0–16: 0 = no histological sign of pulmonary damage and 16 = maximum pulmonary damage. (**f**–**h**) Pulmonary damage scored by histological criteria. Differentiated by IL-6-signaling properties at different time points after trauma.

Posttraumatic alterations in the pulmonary histological examination were apparent after multiple trauma (*p* = 0.017), as well as after isolated bilateral chest trauma (*p* = 0.031), compared to the control (Figure 2e). Examples of the histological features of CNT, as well as after the different trauma types, are shown in Figure 2a–d. In a detailed analysis, we found statistically significant higher scores in FP TT (*p* = 0.010; Figure 2f) compared to FP CNT at 6 h. The microscopic criteria for the pulmonary damage score results were significantly higher in FP TT compared to RKO TT (*p* = 0.014; Figure 2f) at 6 h but otherwise not significantly affected by sgp130Fc and IL-6 receptor knockout.

### 3.2. Cytokines in Blood Plasma

#### 3.2.1. IL-6

**Figure 3:**
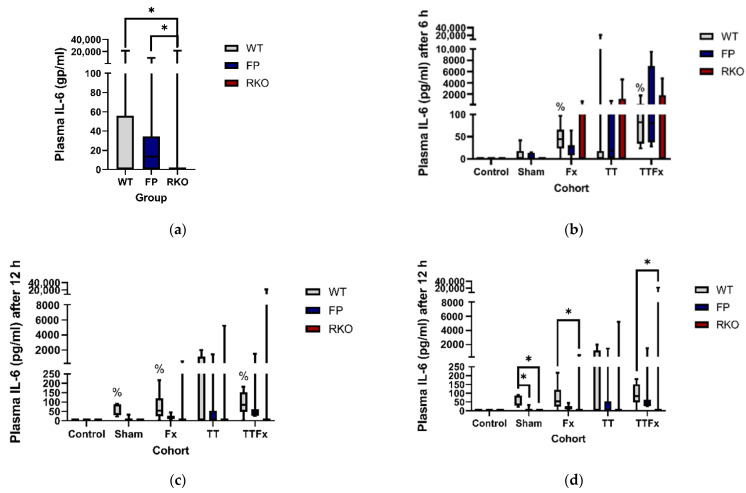

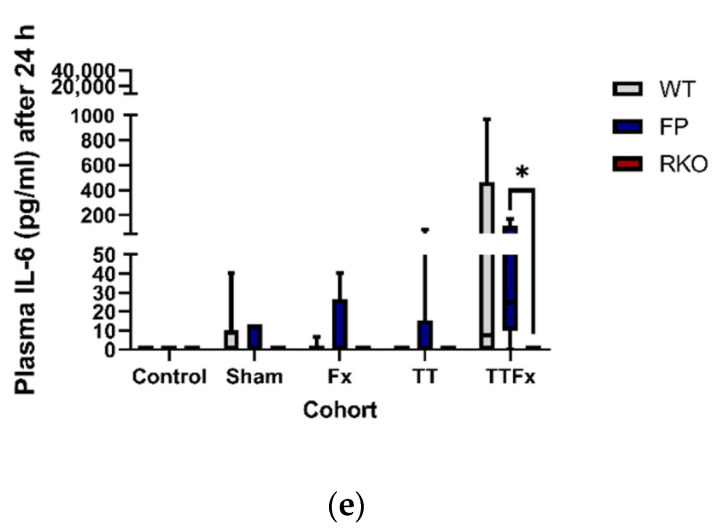
The influence of trauma and IL-6-signaling capacities on the posttraumatic IL-6 plasma levels. Trauma induces an increase in the IL-6 plasma levels. At 12 h after Sham, the IL-6 plasma levels were significantly lower in sgp130Fc-treated (FP) animals compared to untreated wildtype (WT) animals. The IL-6 plasma levels were significantly lower in animals with IL-6 receptor knockout (RKO) than in WT and FP. Control: healthy animals without trauma-generating surgery, Sham: femur pin stabilization, Fx: femoral fracture, TT: bilateral chest trauma and TTFx: bilateral chest trauma plus femoral fracture. *p* < 0.05 * vs. indicated or % vs. Control. (**a**) IL-6 plasma levels compared by different IL-6-signaling capacities. All animals and time points included. (**b**–**e**) Detailed analysis comparing the IL-6 plasma levels in different IL-6-signaling capacities at different time points after the different interventions.

Trauma caused an increase in the IL-6 plasma levels. Including all cohorts and groups, significantly lower IL-6 plasma levels were found in RKO compared to WT (*p* < 0.001) and FP (*p* < 0.001) (Figure 3a). In the WT group, significantly higher IL-6 plasma levels were measured compared to CNT 6 and 12 h after trauma: at 6 h in the Fx (*p* = 0.014; Figure 3b) and TTFx (*p* = 0.03; Figure 3b) cohorts and at 12 h in the SH (*p* = 0.001; Figure 3c), Fx (*p* = 0.006; Figure 3c) and TTFx (*p* = 0.01; Figure 3c) cohorts. In the detailed analysis, significantly lower IL-6 plasma levels were found in RKO compared to WT at 12 h in the SH (*p* = 0.01), Fx (*p* = 0.025) and TTFx (*p* = 0.046) cohorts (Figure 3d). Comparing RKO to FP, significantly lower levels for RKO were found in the TTFx (*p* = 0.005) cohort at 24 h (Figure 3e). The IL-6 plasma levels were found significantly lower in FP compared to the WT at 12 h in the SH (*p* = 0.006) cohort (Figure 3d). 

#### 3.2.2. MCP-3

**Figure 4:**
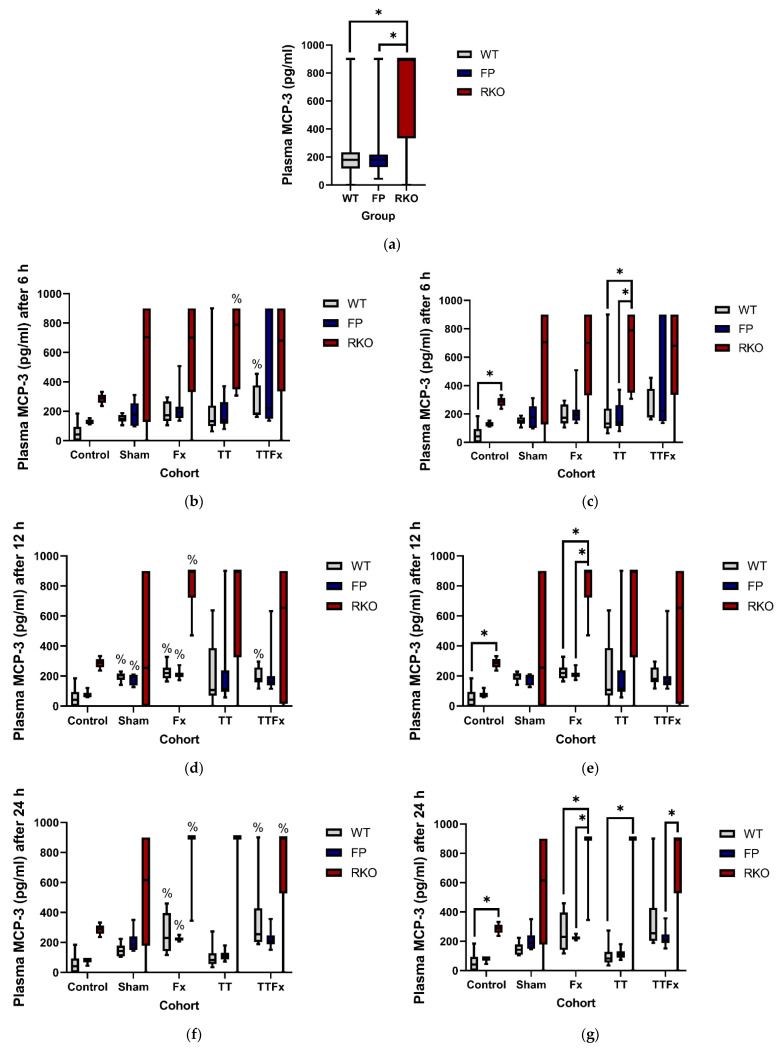
The influence of trauma and IL-6-signaling capacities on the posttraumatic MCP-3 plasma levels. Trauma induces an increase in the MCP-3 plasma levels. The MCP-3 plasma levels were unaffected by the sgp130Fc treatment (FP) but were significantly higher in animals with IL-6 receptor knockout (RKO) than in wildtype (WT) and FP. Control: healthy animals without trauma-generating surgery, Sham: femur pin stabilization, Fx: femoral fracture, TT: bilateral chest trauma and TTFx: bilateral chest trauma plus femoral fracture. *p* < 0.05 * vs. indicated or % vs. Control. (**a**) Posttraumatic MCP-3 plasma levels compared by different IL-6-signaling capacities. All animals and time points included. (**b**–**g**) Detailed analysis comparing the MCP-3 plasma levels in the different IL-6-signaling capacities at different time points after the different interventions.

Trauma caused an increase in the MCP-3 plasma levels. In the WT group, significantly higher MCP-3 plasma levels were measured compared to CNT 6, 12 and 24 h after trauma: at 6 h in the TTFx (*p* = 0.003; Figure 4b) cohort; at 12 h in the SH (*p* = 0.002; Figure 4d), in the Fx (*p* = 0.006; Figure 4d) and in the TTFx (*p* = 0.035; Figure 4d) cohorts and at 24 h in the Fx (*p* = 0.033; Figure 4f) and TTFx (*p* = 0.003; Figure 4f) cohorts. In the FP group, significantly higher MCP-3 plasma levels were measured compared to CNT at 12 h in the SH (*p* = 0.035; Figure 4d) and in the Fx (*p* = 0.008; Figure 4d) cohorts and 24 h in the Fx (*p* = 0.021; Figure 4f) cohort. In the RKO group, significantly higher MCP-3 plasma levels were measured compared to CNT at 6, 12 and 24 h: at 6 h in the TT (*p* = 0.048; Figure 4b) cohort, at 12 h in the Fx (*p* = 0.021; Figure 4d) cohort and at 24 h in the Fx (*p* = 0.004; Figure 4f) and in the TTFx (p = 0.020; Figure 4f) cohorts. 

The MCP-3 baseline (CNT) plasma levels were measured significantly higher in RKO compared to the WT (*p* = 0.005; Figure 4c,e,g). Including all cohorts and groups, significantly higher MCP-3 plasma levels were found in RKO compared to the WT (*p* < 0.001; Figure 4a) and FP (*p* < 0.001; Figure 4a) after trauma. In a detailed analysis, significantly higher MCP-3 plasma levels were measured in RKO compared to the WT at 6 h in the TT (*p* = 0.012; Figure 4c) cohort, at 12 h in the Fx (*p* = 0.004; Figure 4e) cohort and after 24 h in the Fx (*p* = 0.004; Figure 4g) and in the TT (*p* = 0.009; Figure 4g) cohorts. Significantly higher MCP-3 plasma levels were measured in RKO compared to FP at 6 h in the TT (*p* = 0.032; Figure 4c) cohort, at 12 h in the Fx (*p* = 0.001; Figure 4e) cohort and at 24 h in the Fx (*p* = 0.002; Figure 4g) and TTFx (*p* = 0.01; Figure 4g) cohorts. Comparing the WT to FP, no significant difference in the MCP-3 plasma levels was measured. 

#### 3.2.3. RANTES

**Figure 5:**
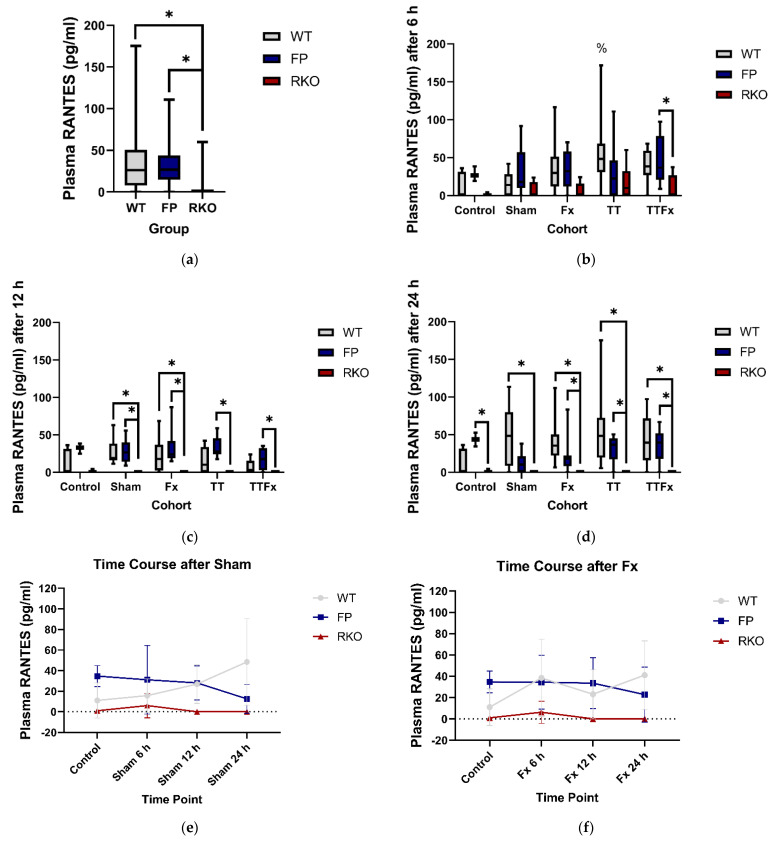

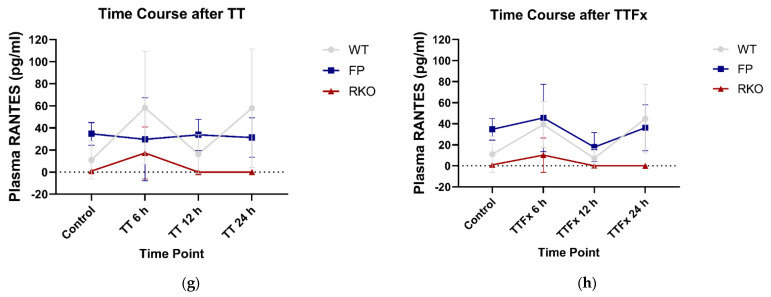
The influence of trauma and IL-6-signaling capacities on the posttraumatic RANTES plasma levels. While the bilateral chest trauma induces an increase in the RANTES plasma levels after 6 h in the wildtype (WT) group, it was no longer detectable in the IL-6 receptor knockout (RKO) group 12 h and 24 h after trauma. In the sgp130Fc-treated animals, RANTES was still detectable at all times of the investigation after trauma. Compared to the WT animals, the plasma RANTES levels tended to be lower in sgp130Fc-treated animals (FP) at the end of our observation period. Control: healthy animals without trauma-generating surgery, Sham: femur pin stabilization, Fx: femoral fracture, TT: bilateral chest trauma and TTFx: bilateral chest trauma plus femoral fracture. *p* < 0.05 * vs. indicated or % vs. Control. (**a**) Posttraumatic RANTES plasma levels compared by different IL-6-signaling capacities. All animals and time points included. (**b**–**d**) Detailed analysis comparing the RANTES plasma levels in the different IL-6-signaling capacities at different time points after the different interventions. (**e**–**h**) RANTES plasma levels at different time points after the respective trauma, distinguished by the different IL-6-signaling capacities.

Trauma caused a significant increase in the RANTES plasma levels compared to CNT in the WT group at 6 h in the TT (*p* = 0.029; Figure 5b) cohort. Including all cohorts and groups, significantly lower RANTES plasma levels were found in RKO compared to the WT (*p* < 0.001; Figure 5a) and FP (*p* < 0.001; Figure 5a). Additionally, in a detailed analysis, the RANTES plasma levels were measured as significantly lower in RKO compared to the WT and in RKO compared to FP at 6, 12 and 24 h after trauma. 

In the control cohorts, RANTES was only detectable in one out of five individuals of the RKO group and in two out of six individuals of the WT group but in all nine individuals of the FP group. There was a subtle increase after trauma. At 6 h, RANTES was detectable in all the trauma cohorts in approximately equivalent values. Comparing the different groups, a significant difference in the RANTES plasma levels was found in the TTFx cohort, with higher levels in the FP group compared to the RKO group (*p* = 0.049; Figure 5b). 

At 12 and 24 h, RANTES was no longer detectable in the trauma cohorts of the RKO group but was still in the WT and FP, leading to significantly lower RANTES plasma levels in RKO compared to the WT and RKO compared to FP in various trauma cohorts: at 12 h, Sham in the WT compared to RKO (*p* = 0.012; Figure 5c), in FP compared to RKO (*p* = 0.006; Figure 5c), in Fx WT compared to RKO (*p* = 0.031; Figure 5c), in FP compared to RKO (*p* = 0.001; Figure 5c) and in TT FP compared to RKO (*p* = 0.001; Figure 5c) and TTFx FP compared to RKO (*p* = 0.005; Figure 5c). 

At 24 h, significantly lower RANTES plasma levels were measured in the RKO group compared to the WT and FP groups: in the CNT cohort in the RKO group compared to the FP group (*p* = 0.025; Figure 5d), in the SH cohort in RKO compared to the WT (*p* = 0.008; Figure 5d), in the Fx cohort in RKO compared to both the WT (*p* < 0.001; Figure 5d) and FP (*p* = 0.033; Figure 5d), in the TT cohort in RKO compared to both the WT (*p* = 0.001; Figure 5d) and FP (*p* = 0.014; Figure 5d) and in the TTFx cohort in RKO compared to the WT (*p* = 0.003; Figure 5d) and FP (*p* = 0.01; Figure 5d). 

After 12 h post-trauma, the plasma RANTES levels remained high in the WT, while they were no longer detectable in RKO (Figure 5e–h), whereas, in FP, the RANTES plasma levels firstly remained comparably high to the WT but tended to moderately decrease towards the end of the observation period. At 24 h, the differences in the plasma RANTES levels between the WT and FP did not reach statistical significance but were consistent throughout all intervention cohorts (Figure 5e–h). 

#### 3.2.4. MCP-1, MIP-1β, GM-CSF 

MCP-1 and MIP-1β were only traceable in a few samples. MCP-1 was neither significantly affected by trauma nor by IL-6 signaling (Supplementary Figure S1). GM-CSF was not traceable in enough plasma samples to generate conclusive results (Supplementary Figure S3). More information can be seen in the Supplementary Materials.

### 3.3. Cytokines in Kupffer Cell Supernatant

#### 3.3.1. MCP-1

**Figure 6:**
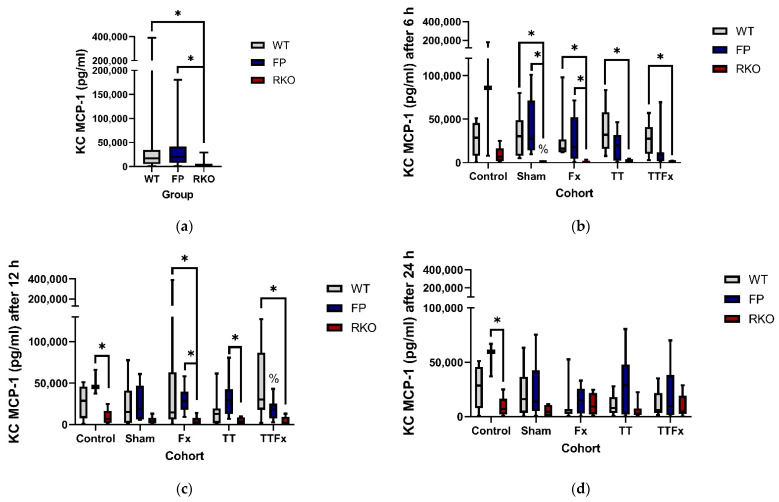
The influence of trauma and IL-6-signaling properties on posttraumatic MCP-1 productive capacities of Kupffer cells (KC). The posttraumatic MCP-1 productive capacity was lower in IL-6 receptor knockout animals (RKO) compared to wildtype animals (WT) and animals treated with sgp130Fc (FP). Control: healthy animals without trauma-generating surgery, Sham: femur pin stabilization, Fx: femoral fracture. TT: bilateral chest trauma and TTFx: bilateral chest trauma plus femoral fracture. *p* < 0.05 * vs. indicated or % vs. Control. (**a**) The posttraumatic MCP-1 productive capacity of KCs, compared by different IL-6-signaling capacities. All animals and time points included. (**b**–**d**) Detailed analysis comparing MCP-1 productive capacities of KCs in the different IL-6-signaling properties at different time points after the different interventions.

Trauma caused a decrease of the MCP-1 productive capacities of Kupffer cells in some cases. In the FP group, a significantly lower MCP-1 productive capacity was measured compared to CNT 12 h after trauma in the TTFx cohort (*p* = 0.028; Figure 6c). In the RKO group, significantly lower MCP-1 productive capacities were measured compared to CNT 6 h after trauma in the SH (*p* = 0.039; Figure 6b) cohort. Including all cohorts and groups, a significantly lower MCP-1 productive capacity was found in RKO compared to the WT (*p* < 0.001; Figure 6a) and FP (*p* < 0.001; Figure 6a) groups. In a detailed analysis, significantly lower MCP-1 productive capacities were found in RKO compared to the WT: at 6 h in the SH (*p* = 0.046; Figure 6b) cohort and in the Fx (*p* = 0.009; Figure 6b), TT (*p* = 0.006; Figure 6b) and TTFx (*p* = 0.006; Figure 6b) cohorts; at 12 h, in the Fx (*p* = 0.049; Figure 6c) and in the TTFx (*p* = 0.002; Figure 6c) cohorts. In a detailed analysis, significantly lower MCP-1 productive capacities were found in RKO compared to FP: at 6 h in the SH (*p* = 0.021; Figure 6b) cohort and in the Fx (*p* = 0.024; Figure 6b) cohort and at 12 h in the Fx (*p* = 0.006; Figure 6c) and the TT (*p* = 0.007; Figure 6c) cohorts. The MCP-1 productive capacity was further measured significantly lower in the RKO group compared to the FP group in CNT at 12 h (*p* = 0.032; Figure 6c) and 24 h (*p* = 0.023; Figure 6d). 

#### 3.3.2. IL-6, MCP-3, RANTES, GM-CSF and MIP-1β

Regarding these cytokines, there were only minor findings concerning the influence of a selective IL-6 trans-signaling inhibition on the posttraumatic cytokine productive capacities of KCs in this study. More information can be seen in the Supplementary Materials. 

### 3.4. Cytokines in Alveolar Macrophages Supernatant

There were only minor findings regarding the influence of a selective IL-6 trans-signaling inhibition on the posttraumatic cytokine productive capacities of AMs in this study. More information can be seen in the Supplementary Materials. 

### 3.5. Cytokines in BAL

There were no relevant findings regarding the cytokine levels in BAL in this study. The different cytokines were either not traceable in enough BAL samples to generate conclusive results or were not significantly influenced by trauma or IL-6-signaling blockades.

## 4. Discussion

The aim of this study was to investigate a potential therapeutic value of a selective inhibition of IL-6 trans-signaling in a posttraumatic course. Therefore, we applied the fusion protein sgp130Fc, an artificial selective IL-6 trans-signaling inhibitor, in a murine multiple trauma model consisting of a femoral fracture and a chest trauma and compared it to wildtype animals with intact IL-6 trans-signaling and interleukin-6 receptor knockout mice. 

Our most important findings are: comparable posttraumatic cytokine levels were seen in the WT and the FP groups. Therefore, no evidence for a major beneficial effect of sgp130Fc on the cytokine release after multiple trauma was detected in this study. However, our trauma model caused severe organ damage and successfully triggered a systemic trauma reaction. Severe organ damage was indicated by posttraumatic alterations in the pulmonary histological examination, which occurred after isolated bilateral chest trauma but also after combined femoral fracture and bilateral chest trauma. An adequate trauma impact, which triggered a systemic posttraumatic immune reaction, was testified by elevated posttraumatic cytokine plasma levels in the WT group. Furthermore, there was a posttraumatic rise in the IL-6 plasma levels, which again supported the role of IL-6 in the posttraumatic immune reaction and suggests that addressing IL-6-meditated processes nonetheless should be the subject of further research. 

Chest traumas and extremity traumas are typical injuries seen in multiple-trauma patients [51]. For our study, we therefore deployed a trauma model representing this potentially life-threatening injury pattern. As a result, we registered an overall mortality of 4.4% in our study. Death occurred directly after the induction of the chest trauma in almost every case. We found internal injuries such as cardiorrhexis, aortic rupture and hemothorax, which can explain the animals’ deaths, for example, due to hemorrhage or obstructive shock. According to Trunkey’s trimodal distribution of trauma deaths [52], the mortality rates in this study can mainly be described as ‘immediate deaths at the scene’. Although we found a slight difference in the mortality comparing the WT to FP, the deaths can be ascribed to direct trauma consequences rather than immunological complications at this early point in time. This study was intended to contribute to answering the question of whether or not a selective inhibition of the IL-6 trans-signaling pathway is conductive to preventing immune-mediated posttraumatic organ failure. For this purpose, an injury severity was required that produced a systemic immune reaction with a liberation of proinflammatory cytokines. A successful establishment of a severe trauma could be deduced from the alterations in the pulmonary histological examination, which occurred after isolated chest trauma but, also, after a combined femoral fracture and bilateral chest trauma. We found statistically significant differences in the pulmonary tissue damage when comparing non-traumatized and traumatized mice. However, we did not see a statistically significant effect in every cohort receiving a bilateral chest trauma at every point in time. Nishina et al. applied the mentioned score before in an acute lung injury by a hydrochloric acid aspiration model in rabbits [50]. They compared the treatment (lidocaine) to no treatment (saline) in aspiration and found statistically significant lower scores in two out of three treatment groups. Unlike us, they instilled fluids for BAL in the right main bronchus and used the left lower lobe for the histological examination. The extent of visible alveolar congestion, hemorrhage and hyaline membranes, as well as the number of intra-alveolar neutrophils, might be influenced by BAL. Unfortunately, BAL itself did not contribute to answering the question of this study. On the downside, the extent of tissue trauma, assessed by the applied score, possibly produced a higher statistical power if BAL was not performed before sample-taking. Possible protective or adverse effects of the sgp130Fc treatment on the degree of pulmonary tissue damage after trauma might have been unrevealed, though existing. This aspect should be considered in further studies. 

Concordant with our histological findings, we registered evidence for a systemic immune reaction regarding the levels of posttraumatic cytokines. Trauma caused an increase of IL-6, RANTES and MCP-3 in the blood plasma in the WT group. This indicates a trauma impact that was competent in stimulating a systemic posttraumatic immune reaction in the organism with full IL-6-signaling capacities. The posttraumatic IL-6 levels were significantly higher after trauma than in the CNT group, which again supports the role of IL-6 in the posttraumatic immune reaction. 

We hypothesized that selective IL-6 trans-signaling inhibition by the therapeutic use of sgp130Fc would beneficially affect the levels of the inflammatory cytokines after multiple trauma. Contrary to our assumption, comparable posttraumatic plasma levels of MCP-3, MCP-1 and RANTES in the WT and the FP groups were found, suggesting that sgp130Fc might not show the expected beneficial effects in the proinflammatory reaction in this setting. Additionally, posttraumatic cytokine productive capacities of KCs and AMs did not produce groundbreaking findings. Except reduced MCP-1 and MIP-1β productive capacities of AMs in animals treated with sgp130 Fc compared to WT, the cytokine productive capacities were either unaffected or, in the case of MCP-3, even elevated in FP. 

Although the effect on the posttraumatic cytokine release fell short of expectations, we still detected it. At 12 h, in the SH cohort, we detected significantly lower IL-6 plasma levels in the animals treated with sgp130Fc than in the untreated animals with full IL-6-signaling capacities. No further systemic cytokine reduction was seen associated with the sgp130Fc treatment in the other trauma groups or at different times to provide stronger evidence. 

A possible explanation may lie in the sgp130Fc dosing. Herein, we applied a dose of 0.5 mg/kg. Barkhausen et al. [40] found a dose optimum at 0.5–2.5 mg/kg for the preventive use of sgp130Fc in a murine cecal ligation and puncture polymicrobial sepsis model. Therein, a pretherapy with 0.5-mg/kg sgp130Fc 24 h prior to the intervention significantly improved the survival after polymicrobial sepsis. Applying lower doses did not improve the survival. A preventive approach does not appear applicable to trauma. However, an increase in the dosage may help to amplify a potential beneficial effect of the therapeutic utilization post-trauma. However, it should be taken into account that very high sgp130Fc dosages can also inhibit IL-6 classic signaling by trapping major quantities of IL-6 in a IL-6-sIL-6R–sgp130Fc complex [53]. Barkhausen et al. [40] also investigated the therapeutic potential of sgp130Fc in the same model. Therefore, they applied a dose of 1 mg/kg 24 h after the intervention, which also increased survival, albeit not to statistical significance. These findings suggest that a moderately higher dose of 1–2.5 mg/kg might result in a higher therapeutic value.

Kaiser et al. [25] applied sgp130Fc in a comprised fracture healing model, also consisting of a chest trauma and femoral fractur. Therein, also, a dosage of 0.5-mg/kg sgp130Fc was applied in close temporal relation (30 min after induction) to the injury. They found reduced levels of circulating IL-6 after 3 h but increased levels after one day. Similar to our study, there was no major effect of the sgp130Fc treatment on the degree of pulmonary damage. Nevertheless, they assessed an improvement in fracture healing after sgp130Fc treatment, indicating its beneficial effects in the postinjury organism. Unlike in our study, the observation period was longer (up to 21 days), and a second dose of sgp130Fc was administered 48 h after injury. Just as an increase of the dosing, also a dose repetition might have effectuated a stronger limitation of proinflammatory immune reactions after trauma in this study. 

Nevertheless, hints for a coordinated immune modulation due to a selective IL-6-signaling inhibition appear to exist regarding the posttraumatic levels of the cytokine RANTES. After an initial trauma-dependent increase, the levels remained high in the WT, while the levels drastically decreased in RKO, whereas, in FP, after the initial increase, the levels firstly remained comparably high to the WT but tended to moderately decrease over the course of time. Since RANTES is not only suspected to have a role in inflammatory disorders [54] but shows contributing effects in bone formation and remodeling [55], the opportunity to finely tune levels by selective IL-6 signaling inhibition seems rewarding. Unfortunately, this effect was not strong enough to produce statistical significance. Since our data suggested a time-dependent course of plasma RANTES levels under sgp130Fc influence, a longer observation period that could be combined with dose repetition might supply significant data regarding RANTES and, potentially, even result in the inflammation-limiting effect we initially hypothesized.

### Limitations and Strength

For more pronounced microscopic results after trauma, histological lung samples should be taken out of the tissue that has not been undergoing BAL. There is evidence that gender influences the incidence of MODS in multiple-injured patients [56]. Additionally, gender-specific differences regarding inflammatory cytokine production have been demonstrated for ischemia/reperfusion [57] and hemorrhage models [58]. Therefore, further studies with female mice are required. In order to enhance the understanding of trauma immunological processes in later stages postinjury, further research needs to be conducted. In this study, we deployed sgp130Fc only in one dosage (0.5 mg/kg) at one point of time. Further studies are required to disclose the effects of other dosages and dose repetition on trauma-induced cytokine releases. Since this study focused on the influence of sgp130Fc on microscopic pulmonary changes and specific cytokine levels after trauma, more investigations regarding further inflammatory consequences of the IL-6 trans-signaling inhibition are needed. The trauma model applied in this study successfully triggered a systemic trauma reaction, which can provide the basis for further studies, for example, to investigate sgp130Fc at different dosages after multiple trauma.

## 5. Conclusions

Consulting the microscopic criteria, there was no protective effect of sgp130Fc on the degree of pulmonary contusion. Comparable cytokine levels in the WT and FP indicated neither a strong protective nor an adverse effect of a single dosage of sgp130Fc on the cytokine release after femoral fracture and bilateral chest trauma.

## Data Availability

The original data of this study can be received from the corresponding author by reasonable request.

## Supplementary Materials

The following are available online at https://www.mdpi.com/article/10.3390/life11111252/s1, Figure S1: The influence of trauma and IL-6 signaling capacities on the posttraumatic MCP-1 plasma levels; Figure S2: The influence of trauma and IL-6 signaling capacities on the posttraumatic MIP-1β plasma levels; Figure S3: The influence of trauma and IL-6 signaling capacities on the posttraumatic GM-CSF plasma levels; Figure S4: The influence of trauma and IL-6 signaling properties on the posttraumatic IL-6 productive capacities of Kupffer cells (KC); Figure S5: The influence of trauma and IL-6 signaling properties on the posttraumatic MCP-3 productive capacities of Kupffer cells (KC); Figure S6: The influence of trauma and IL-6 signaling properties on the posttraumatic RANTES productive capacities of Kupffer cells (KC); Figure S7: The influence of trauma and IL-6 signaling properties on the posttraumatic GM-CSF productive capacities of Kupffer cells (KC); Figure S8: The influence of trauma and IL-6 signaling properties on the posttraumatic MIP-1β productive capacities of Kupffer cells (KC); Figure S9: The influence of trauma and IL-6 signaling properties on the posttraumatic IL-6 productive capacities of alveolar macrophages (AM); Figure S10: The influence of trauma and IL-6 signaling properties on the posttraumatic MCP-3 productive capacities of alveolar macrophages (AM); Figure S11: The influence of trauma and IL-6 signaling properties on the posttraumatic MCP-1 productive capacities of alveolar macrophages (AM); Figure S12: The influence of trauma and IL-6 signaling properties on the posttraumatic RANTES productive capacities of alveolar macrophages (AM); Figure S13: The influence of trauma and IL-6 signaling properties on the posttraumatic GM-CSF productive capacities of alveolar macrophages (AM); Figure S14: The influence of trauma and IL-6 signaling properties on the posttraumatic MIP-1β productive capacities of alveolar macrophages (AM). Table S1: Group distribution.
