# Supplementary material for: Selective Inhibition of IL-6 Trans-Signaling Has No Beneficial Effect on the Posttraumatic Cytokine Release after Multiple Trauma in Mice
Source: Life (Basel). 2021 Nov 17;11(11):1252. doi: 10.3390/life11111252 (PMC8617644; doi:10.3390/life11111252)
Supplement: Supplementary file 1 [file life-11-01252-s001.zip › life-1444434-supplementary.pdf]

life

MDPI

Supplementary Material of Selective Inhibition of IL-6 Trans-Signaling Has no Beneficial Effect on the Posttraumatic Cytokine Release after Multiple Trauma in Mice

1. Supplementary Materials

1.1. Group Distribution

Table S1. Group distribution. For this study we compared wildtype mice with full IL-6 signaling capacities to wildtype mice with inhibited IL-6 trans-signaling and IL-6 receptor knockout mice at 6 h, 12 h and 24 h after different traumas. Control: healthy animals without trauma generating surgery; Sham: femur pin stabilization; Fx: femoral fracture; TT: bilateral chest trauma, TTFx: bilateral chest trauma plus femoral fracture.

**Cohort (Procedure)**	**Wildtype (WT) (n)**				**Wildtype + sgp130Fc (FP) (n)**				**IL-6R knockout (RKO) (n)**			
	**0 h**	**6 h**	**12 h**	**24 h**	**0 h**	**6 h**	**12 h**	**24 h**	**0 h**	**6 h**	**12 h**	**24 h**
Control (no intervention)	6					3	3	3	5			
Sham (pin stabilization)		6	6	6		6	6	6		4	6	6
Fx (pin stabilization + femoral fracture)		8	8	8		8	8	8		6	8	8
TT (bilateral chest trauma)		8	8	8		8	8	8		6	8	8
TTFx (pin stabilization + femoral fracture + bilateral chest trauma)		8	8	9		8	8	8		6	8	8

1.2. Cytokines in Blood Plasma

1.2.1 MCP-1, MIP-1β, GM-CSF

MCP-1, MIP-1β and GM-CSF were only traceable in few samples, impeding the statistical analysis. MCP-1 was neither by trauma nor by IL-6 signaling significantly affected (Supp. Fig. 1). Regarding MIP-1β, there were some statistical results, however the number of positive samples was very small and conclusive results are doubtful. MIP-1β plasma levels were statistically higher in the CNT cohort in FP compared to WT at 12 h ($p = 0.049$; Figure S2(**c**)) and to WT at 24 h ($p = 0.037$; Figure S2(**d**)) and RKO at 24 h ($p = 0.027$; Supp. Figure S2(**d**)). At 12 h in FP the MIP-1β plasma levels were significantly lower in the TT ($p = 0.027$; Supp. Fig. 2(**c**)) cohort compared to the CNT cohort, at 24 h in the SH ($p = 0.002$; Figure S2(**d**)) cohort compared to the CNT cohort. GM-CSF was not traceable in enough plasma samples to generate conclusive results (Figure S3).

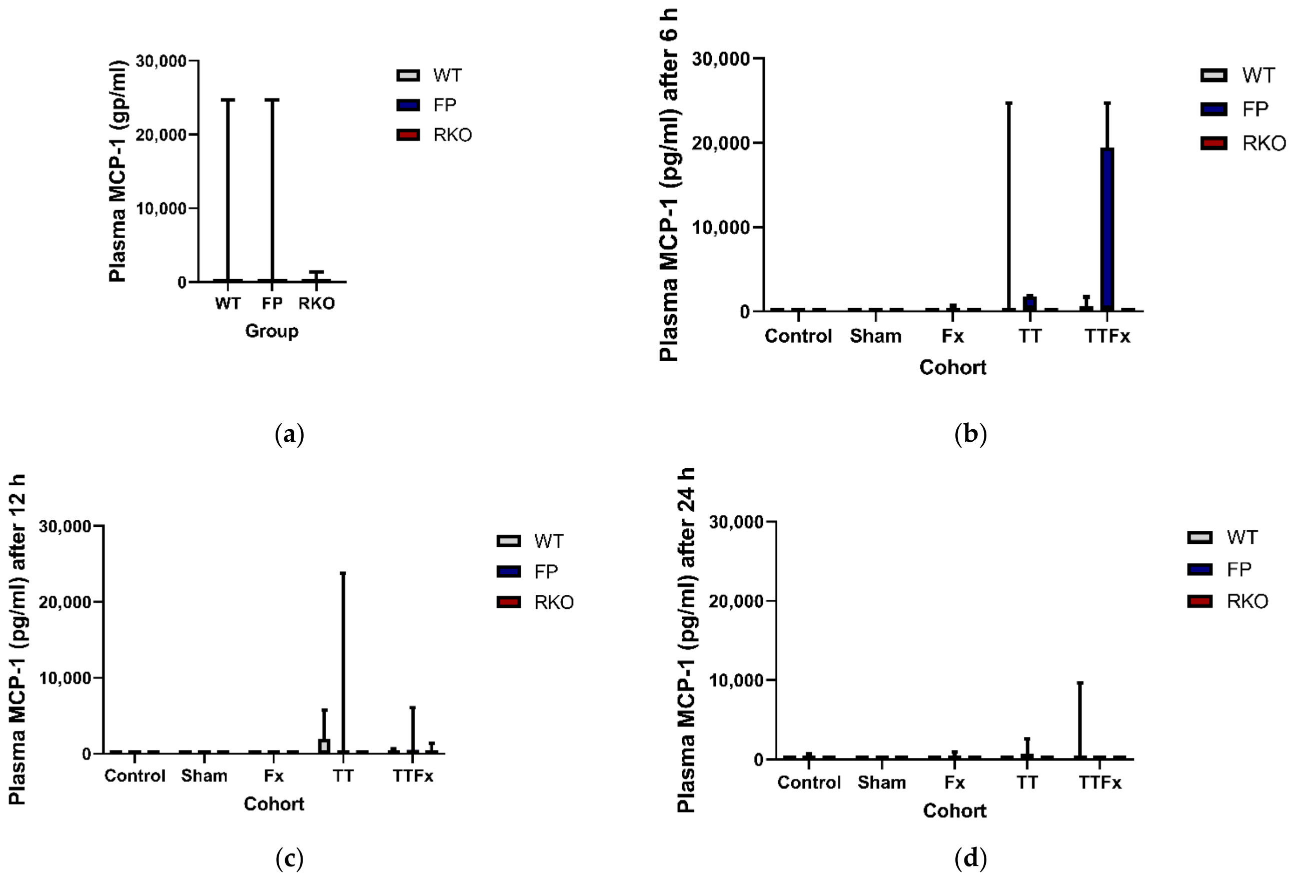

Figure S1. The influence of trauma and IL-6 signaling capacities on the posttraumatic MCP-1 plasma levels. MCP-1 was traceable only in few samples. There were no major differences in MCP-3 plasma levels comparing untreated wildtype animals (WT), animals treated with sgp130Fc (FP) and animals with IL-6 receptor knockout (RKO). Control: healthy animals without trauma generating surgery; Sham: Femur pin stabilization; Fx: femoral fracture; TT: bilateral chest trauma, TTFx: bilateral chest trauma plus femoral fracture. $p < 0.05$ * *vs.* indicated or % *vs.* Control. (**a**) Posttraumatic MCP-3 plasma levels compared by different IL-6 signaling capacities. All animals and time points included. (**b**–**d**) Detailed analysis comparing MCP-3 plasma levels in the different IL-6 signaling capacities at different time points after the different interventions.

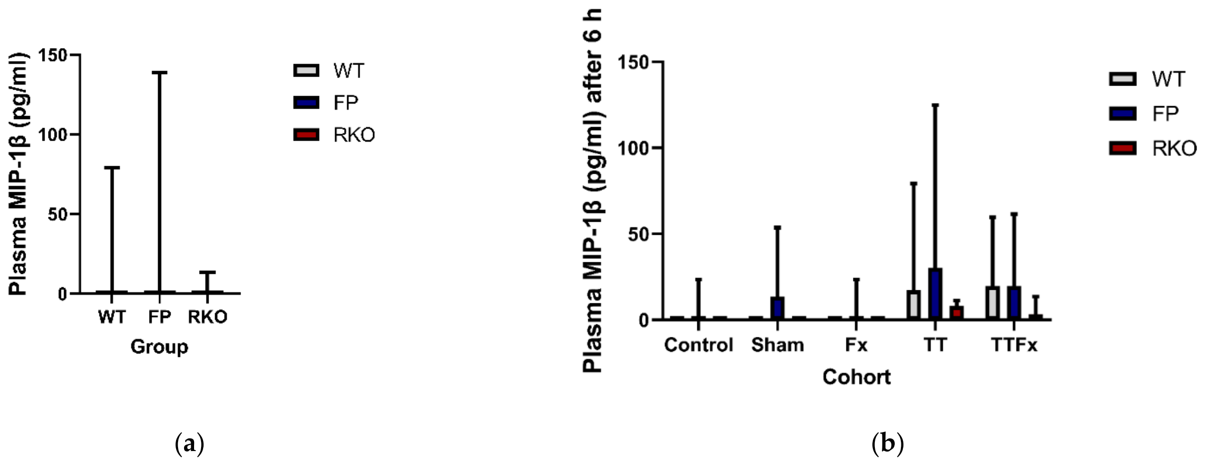

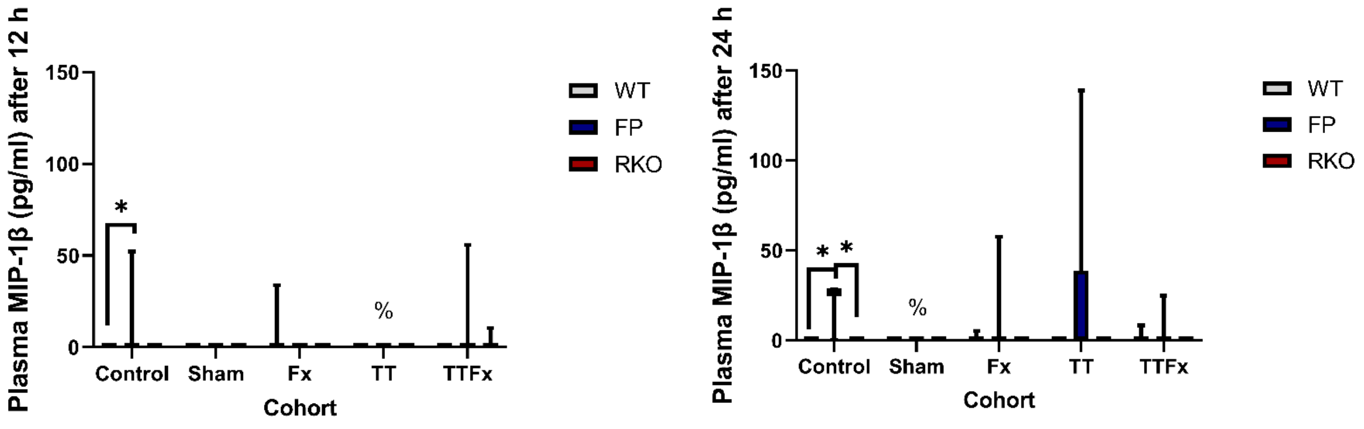

(**c**) (**d**)

Figure S2. The influence of trauma and IL-6 signaling capacities on the posttraumatic MIP-1β plasma levels. MIP-1β was traceable only in few samples, impeding the statistical analysis. Besides differences in the CNT cohorts, there were no major differences in MIP-1β plasma levels comparing untreated wildtype animals (WT), animals treated with sgp130Fc (FP) and animals with IL-6 receptor knockout (RKO). Control: healthy animals without trauma generating surgery; Sham: Femur pin stabilization; Fx: femoral fracture; TT: bilateral chest trauma, TTFx: bilateral chest trauma plus femoral fracture. $p < 0.05$ * *vs.* indicated or % *vs.* Control. (**a**) Posttraumatic MIP-1β plasma levels compared by different IL-6 signaling capacities. All animals and time points included. (**b**–**d**) Detailed analysis comparing MIP-1β plasma levels in the different IL-6 signaling capacities at different time points after the different interventions.

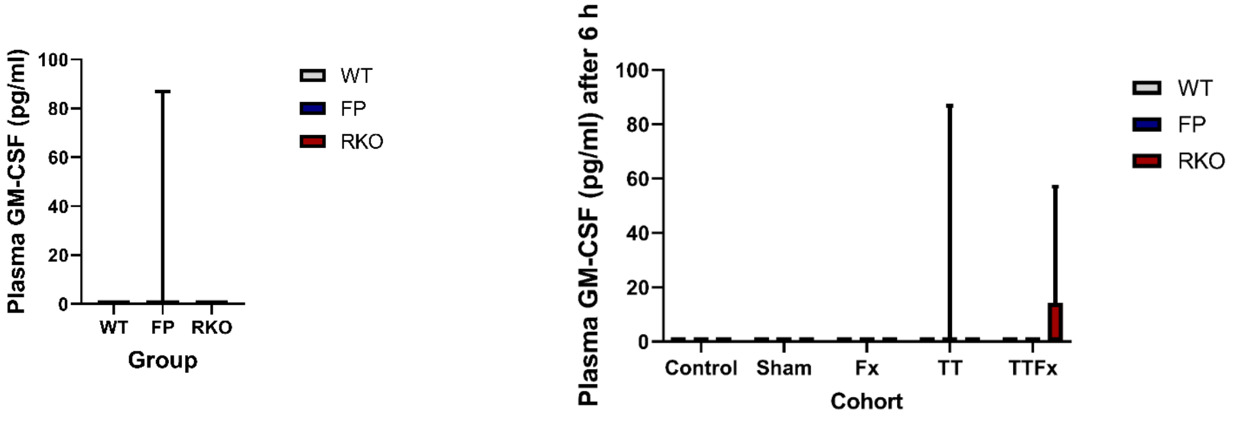

(**a**) (**b**)

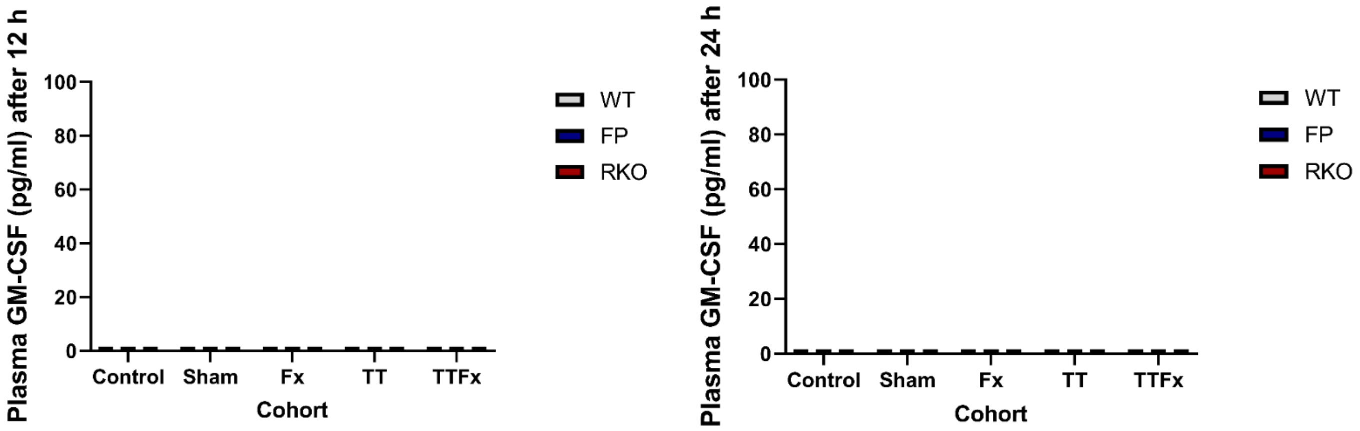

(**c**) (**d**)

Figure S3. The influence of trauma and IL-6 signaling capacities on the posttraumatic GM-CSF plasma levels. GM-CSF was traceable in almost no sample. Analysis was not applicable to investigate for differences in GM-CSF plasma levels

comparing untreated wildtype animals (WT), animals treated with sgp130Fc (FP) and animals with IL-6 receptor knockout (RKO). Control: healthy animals without trauma generating surgery; Sham: Femur pin stabilization; Fx: femoral fracture; TT: bilateral chest trauma, TTFx: bilateral chest trauma plus femoral fracture. $p < 0.05$ * *vs.* indicated or % *vs.* Control. (**a**) Posttraumatic GM-CSF plasma levels compared by different IL-6 signaling capacities. All animals and time points included. (**b**-**d**) Detailed analysis comparing GM-CSF plasma levels in the different IL-6 signaling capacities at different time points after the different interventions.

1.3. Cytokines in Kupffer Cell Supernatant

1.3.1. IL-6

Trauma caused a decrease of the IL-6 productive capacities of Kupffer cells in some cases. In the FP group significantly reduced IL-6 productive capacities were measured compared to CNT: at 12 h in the TTFx (p = 0.029; Figure S4(**c**)) cohort and at 24 h in the TTFx (p = 0.032; Figure S4(**d**)) cohort. In the RKO group significantly reduced IL-6 productive capacities were measured compared to CNT: at 6 h in the SH (p = 0.043; Figure S4(**b**)) and the Fx (p = 0.038; Figure S4(**b**)) cohort. There were no significant differences in the IL-6 productive capacity of Kupffer cells comparing WT, FP and RKO.

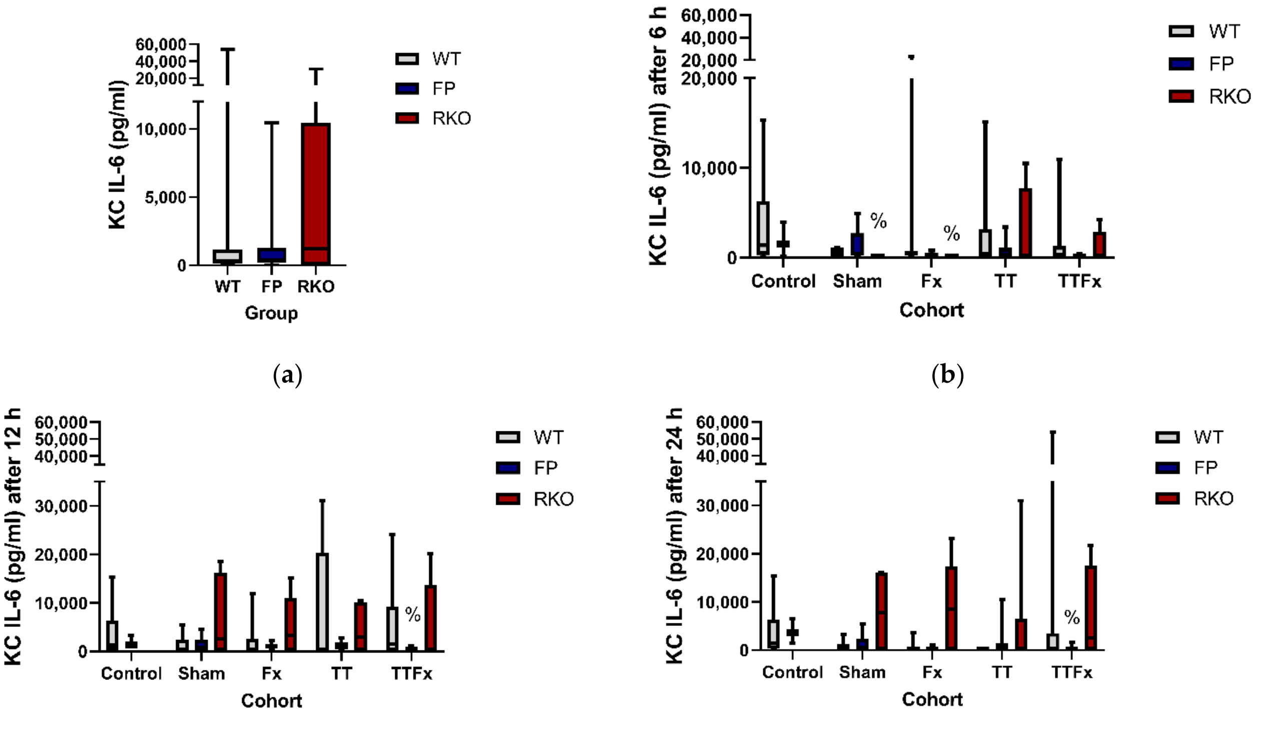

(**a**) (**b**) (**c**) (**d**)

Figure S4. The influence of trauma and IL-6 signaling properties on the posttraumatic IL-6 productive capacities of Kupffer cells (KC). Trauma caused a decrease of the IL-6 productive capacities of Kupffer cells in some cases. There was no significant difference regarding the IL-6 productive capacity of KCs in animals treated with sgp130Fc (FP), compared to untreated wildtype animals (WT) and IL-6 receptor knockout animals (RKO). Control: healthy animals without trauma generating surgery; Sham: Femur pin stabilization; Fx: femoral fracture; TT: bilateral chest trauma, TTFx: bilateral chest trauma plus femoral fracture. $p < 0.05$ * *vs.* indicated or % *vs.* Control. (**a**) The posttraumatic IL-6 productive capacity of KCs, compared by different IL-6 signaling capacities. All animals and time points included. (**b**–**d**) Detailed analysis comparing IL-6 productive capacities of KCs in the different IL-6 signaling properties at different time points after the different interventions.

1.3.2. MCP-3

Trauma caused a decreased MCP-3 productive capacity in Kupffer cells in some cases. In the FP group significantly lower MCP-3 productive capacities were measured compared to CNT after trauma: at 12 h in the TTFx cohort (p = 0.035; Figure S5 (**c**)) and at

24 h in the Fx (p = 0.030; Figure S5(**d**)) cohort. There was no significant difference in the MCP-3 productive capacity comparing WT, FP and RKO.

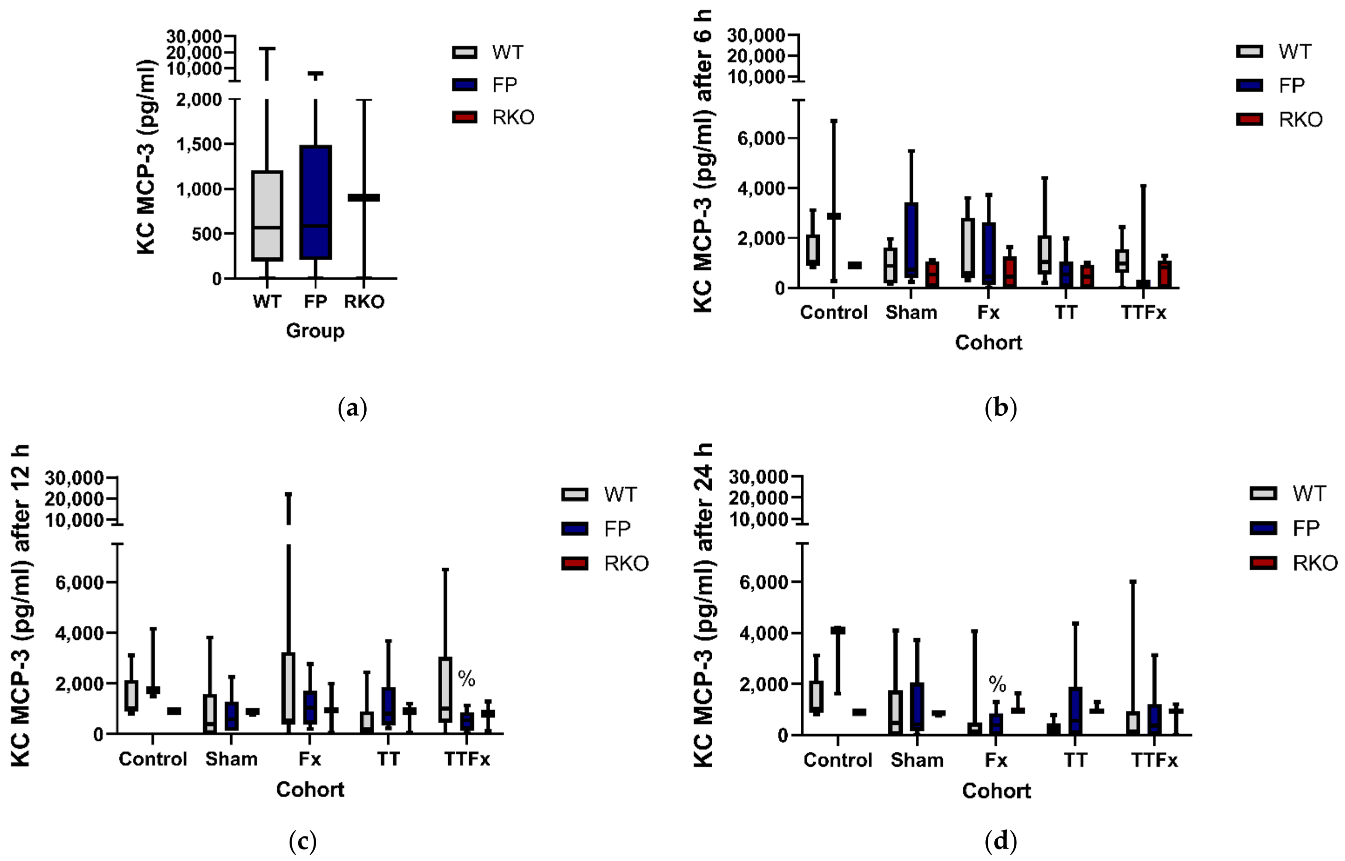

Figure S5. The influence of trauma and IL-6 signaling properties on the posttraumatic MCP-3 productive capacities of Kupffer cells (KC). Trauma caused a decrease of the IL-6 productive capacities of Kupffer cells in some cases. There was no significant difference in the MCP-3 productive capacity of KCs in animals treated with sgp130Fc (FP), compared to untreated wildtype animals (WT) and IL-6 receptor knockout animals (RKO), Control: healthy animals without trauma generating surgery; Sham: Femur pin stabilization; Fx: femoral fracture; TT: bilateral chest trauma, TTFx: bilateral chest trauma plus femoral fracture. $p < 0.05$ * *vs.* indicated or % *vs.* Control. (**a**) The posttraumatic MCP-3 productive capacity of KCs, compared by different IL-6 signaling capacities. All animals and time points included. (**b**–**d**) Detailed analysis comparing MCP-3 productive capacities of KCs in the different IL-6 signaling properties at different time points after the different interventions.

1.3.4. RANTES

Trauma mainly did not affect the RANTES productive capacity. We found a significantly lower productive capacity compared in the RKO group at 6 h, comparing Fx (p = 0.026; Figure S6(**b**)). There were no significant differences in the RANTES productive capacity of Kupffer cells comparing WT, FP and RKO.

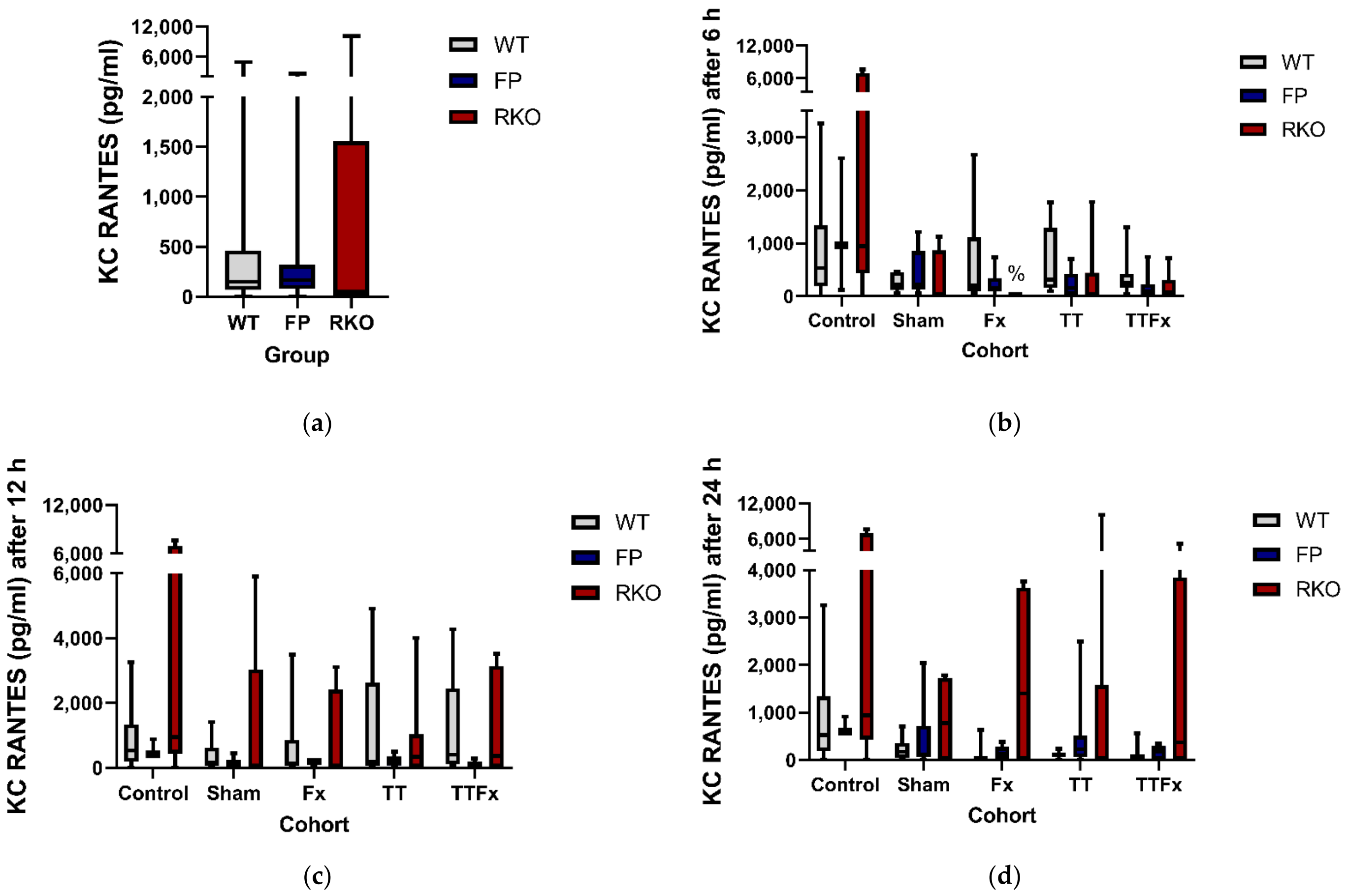

Figure S6. The influence of trauma and IL-6 signaling properties on the posttraumatic RANTES productive capacities of Kupffer cells (KC). Trauma mainly did not affect the RANTES productive capacity. There were no significant differences in the RANTES productive capacity of KCs in animals treated with sgp130Fc (FP), compared to untreated wildtype animals (WT) and IL-6 receptor knockout animals (RKO). Control: healthy animals without trauma generating surgery; Sham: Femur pin stabilization; Fx: femoral fracture; TT: bilateral chest trauma, TTFx: bilateral chest trauma plus femoral fracture. $p < 0.05$ * *vs.* indicated or % *vs.* Control. (**a**) The posttraumatic RANTES productive capacity of KCs, compared by the different IL-6 signaling capacities. All animals and time points included. (**b**–**d**) Detailed analysis comparing RANTES productive capacities of KCs in the different IL-6 signaling properties at different time points after the different interventions.

1.3.5. GM-CSF

Trauma mainly did not affect GM-CSF productive capacity in KCs. A significantly lower GM-CSF productive capacity was found in the TTFx cohort compared to CNT in the FP group at 24 h ($p = 0.040$; Figure S7(**d**)). GM-CSF productive capacities were otherwise unaffected by the trauma. Including all cohorts and groups, a significantly lower GM-CSF productive capacity was found in RKO compared to FP ($p < 0.001$; Figure S7(**a**)). In detailed analysis, we found no significant differences in the GM-CSF productive capacity comparing WT, FP and RKO in the different trauma cohorts.

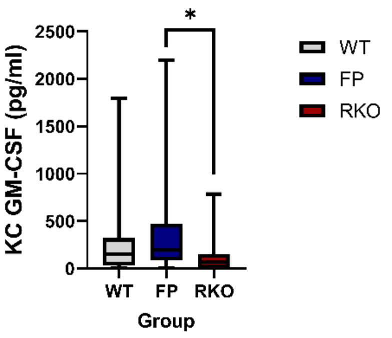

(**a**)

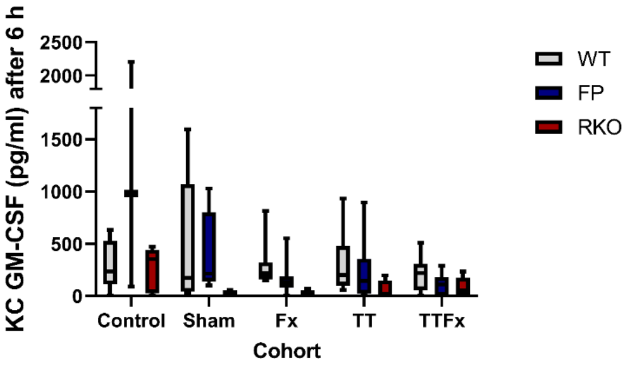

(**b**)

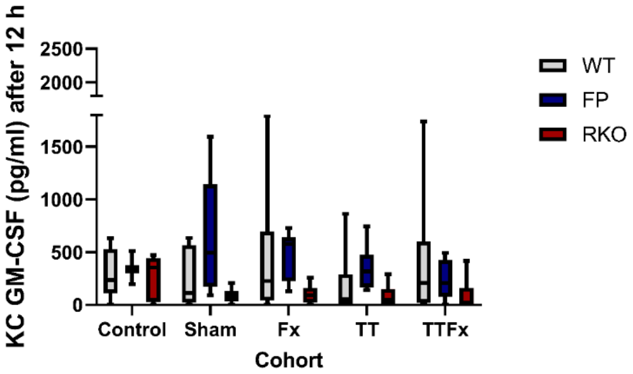

(**c**)

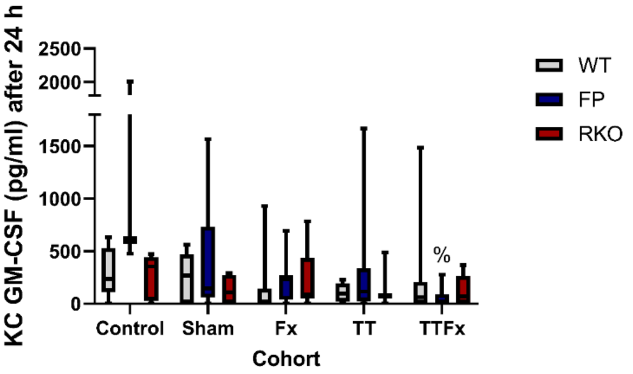

(**d**)

Figure S7. The influence of trauma and IL-6 signaling properties on the posttraumatic GM-CSF productive capacities of Kupffer cells (KC). Trauma mainly did not affect GM-CSF productive capacity in KCs. There were no significant differences in the RANTES productive capacity of KCs in animals treated with sgp130Fc (FP), compared to untreated wildtype animals (WT) and IL-6 receptor knockout animals (RKO). Control: healthy animals without trauma generating surgery; Sham: Femur pin stabilization; Fx: femoral fracture; TT: bilateral chest trauma, TTFx: bilateral chest trauma plus femoral fracture. $p < 0.05$ * *vs.* indicated or % *vs.* Control. (**a**) The posttraumatic GM-CSF productive capacity of KCs, compared by the different IL-6 signaling capacities. All animals and time points included. A significantly lower GM-CSF productive capacity was found in RKO compared to FP. (**b**–**d**) Detailed analysis comparing GM-CSF productive capacities of KCs in the different IL-6 signaling properties at different time points after the different interventions.

3.3.6. MIP-1β

Trauma did not affect MIP-1β productive capacities. There were no significant differences in the MIP-1β productive capacity of Kupffer cells comparing WT, FP and RKO.

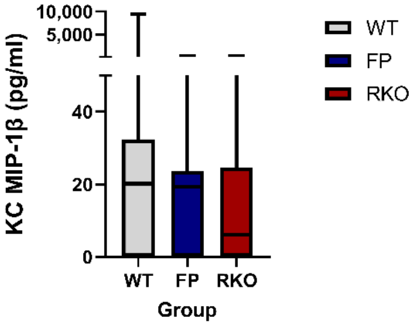

(**a**)

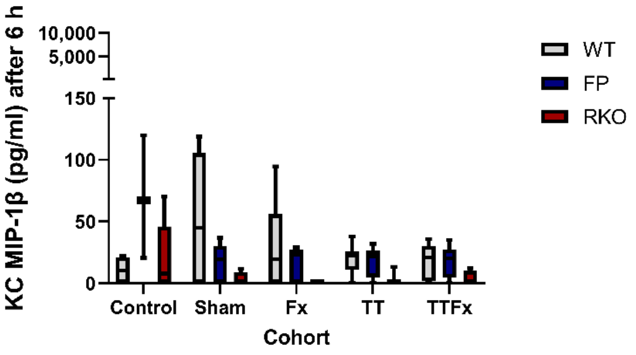

(**b**)

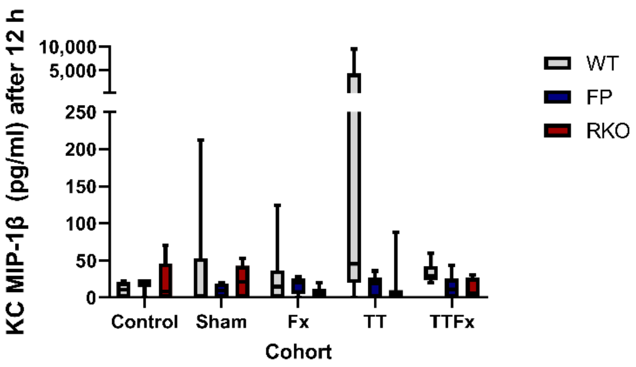

(**c**)

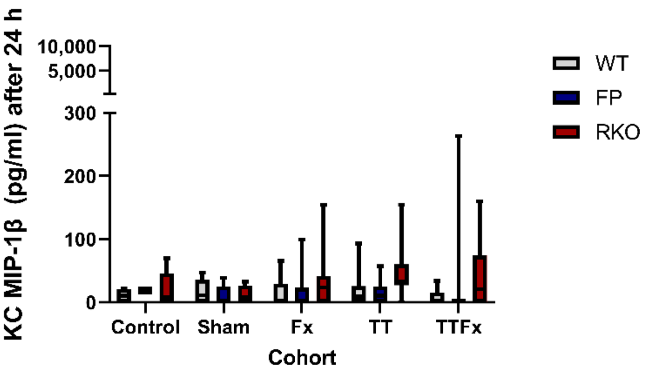

(**d**)

Figure S8. The influence of trauma and IL-6 signaling properties on the posttraumatic MIP-1β productive capacities of Kupffer cells (KC). Trauma did not affect MIP-1β productive capacity in KCs. There were no significant differences in the MIP-1β productive capacity of KCs in animals treated with sgp130Fc (FP), compared to untreated wildtype animals (WT) and IL-6 receptor knockout animals (RKO). Control: healthy animals without trauma generating surgery; Sham: Femur pin stabilization; Fx: femoral fracture; TT: bilateral chest trauma, TTFx: bilateral chest trauma plus femoral fracture. $p < 0.05$ * *vs.* indicated or % *vs.* Control. (**a**) The posttraumatic MIP-1β productive capacity of KCs, compared by the different IL-6 signaling capacities. All animals and time points included. A significantly lower MIP-1β productive capacity was found in RKO compared to FP. (**b**–**d**) Detailed analysis comparing MIP-1β productive capacities of KCs in the different IL-6 signaling properties at different time points after the different interventions. No significant differences found.

1.4. Cytokines in Alveolar Macrophages

1.4.1. IL-6

Trauma did not affect IL-6 productive capacity in AMs. Including all cohorts and groups, a significantly lower IL-6 productive capacity was found in FP compared to RKO ($p = 0.006$; Figure S9(**a**)). In detailed analysis, we found no significant differences in the IL-6 productive capacity comparing WT, FP and RKO in the different trauma cohorts.

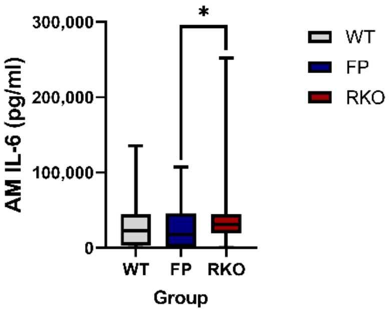

(**a**)

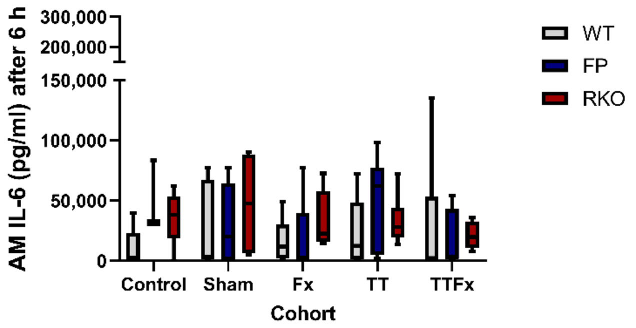

(**b**)

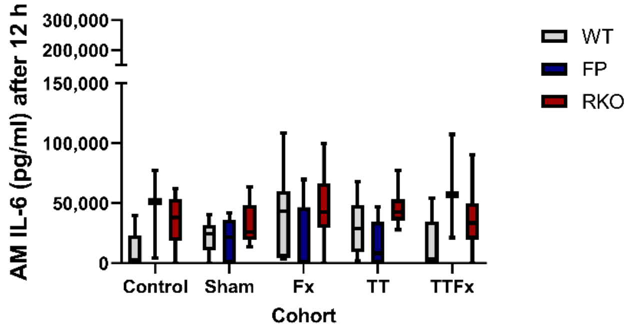

(**c**)

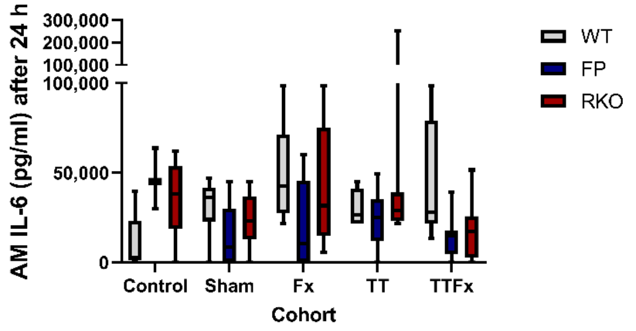

(**d**)

Figure S9. The influence of trauma and IL-6 signaling properties on the posttraumatic IL-6 productive capacities of alveolar macrophages (AM). Trauma did not affect IL-6 productive capacity in AMs. Only while including all cohorts and groups, a significantly lower IL-6 productive capacity was found in animals treated with sgp130Fc (FP), compared to IL-6 receptor knockout animals (RKO). There were no significant differences compared to untreated wildtype animals. Control: healthy animals without trauma generating surgery; Sham: Femur pin stabilization; Fx: femoral fracture; TT: bilateral chest trauma, TTFx: bilateral chest trauma plus femoral fracture. $p < 0.05$ * *vs.* indicated or % *vs.* Control. (**a**) The posttraumatic IL-6 productive capacity of AMs, compared by the different IL-6 signaling capacities. All animals and time points included. (**b–d**) Detailed analysis comparing IL-6 productive capacities of AMs in the different IL-6 signaling properties at different time points after the different interventions. No significant differences found.

1.4.2. MCP-3

Trauma did not affect MCP-3 productive capacities, except in one case: We found a significantly lower MCP-3 productive capacity after trauma at 12 h in the FP group, comparing the TT cohort to CNT (p = 0.007; Figure S10(**c**)). Including all cohorts and groups, a significantly lower MCP-3 productive capacity was found in FP compared to WT (p = 0.002; Figure S10(**a**)) and RKO (p < 0.001; Figure S10(**a**)). In detailed analysis significant differences in MCP-3 productive capacities were only found in the CNT cohorts at 12 h with significantly higher MCP-3 productive capacities in FP compared to WT (p = 0.031; Figure S10(**c**)) and RKO compared to WT (p = 0.044; Figure S10(**c**)), as well as significantly lower MCP-3 productive capacities in the TT cohort comparing FP to RKO (p = 0.007; Figure S10(**c**)).

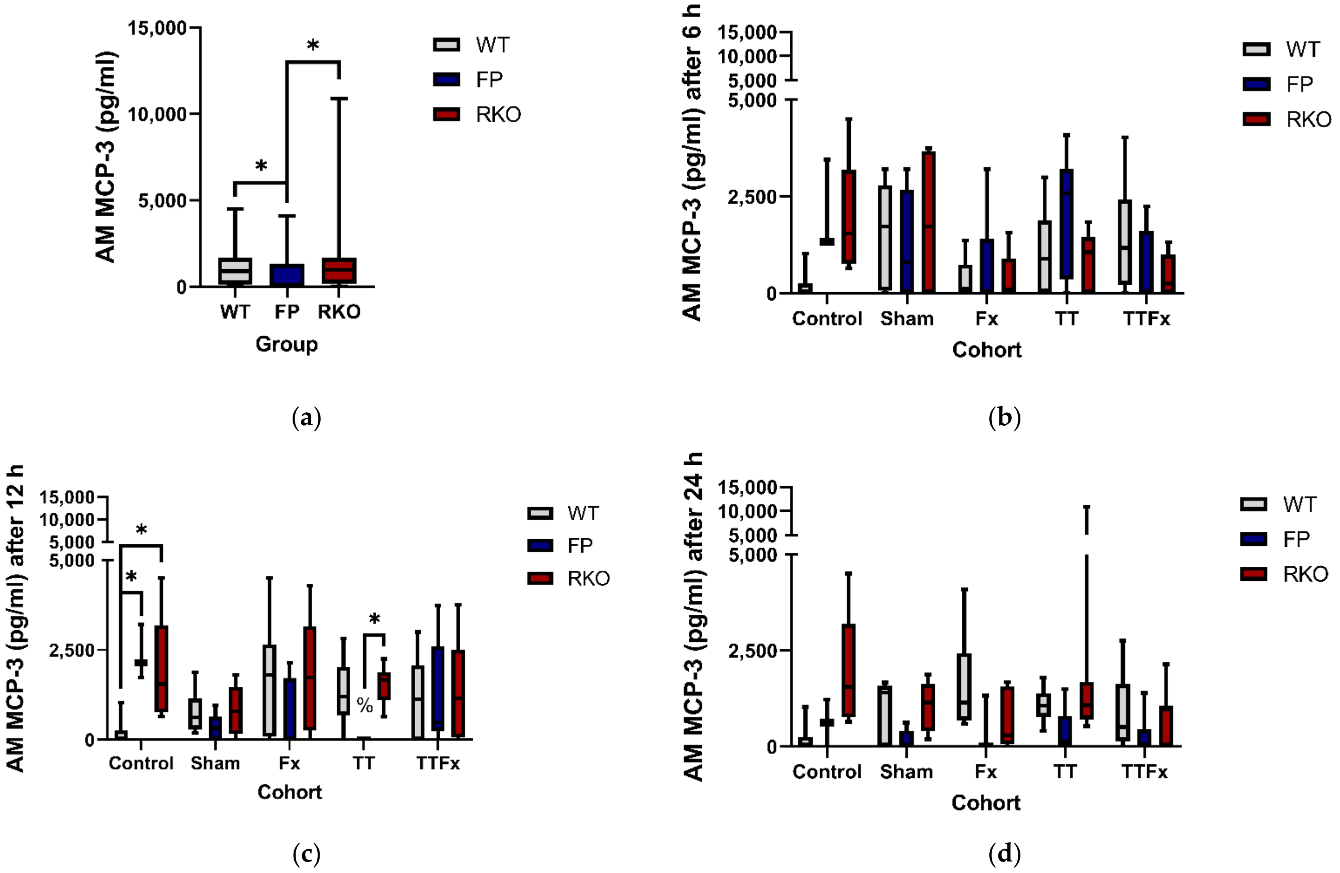

Figure S10. The influence of trauma and IL-6 signaling properties on the posttraumatic MCP-3 productive capacities of alveolar macrophages (AM). Trauma did only affect MCP-3 productive capacities of AMs, in the FP group in the TT cohort at 12 h. Including all cohorts and groups, a significantly lower MCP-3 productive capacity was found in animals treated with sgp130Fc (FP), compared to IL-6 receptor knockout animals (RKO) and untreated wildtype animals. Control: healthy animals without trauma generating surgery; Sham: Femur pin stabilization; Fx: femoral fracture; TT: bilateral chest trauma, TTFx: bilateral chest trauma plus femoral fracture. $p < 0.05$ * *vs.* indicated or % *vs.* Control. (**a**) The posttraumatic MCP-3 productive capacity of AMs, compared by the different IL-6 signaling properties. All animals and time points

included. (**b**–**d**) Detailed analysis comparing MPC-3 productive capacities of AMs in the different IL-6 signaling properties at different time points after the different interventions.

1.4.3. MCP-1

Trauma did not significantly influence the MCP-1 productive capacity in alveolar macrophages. Including all cohorts and groups, a significantly higher MCP-1 productive capacity was found in RKO compared to WT (p = 0.003; Figure S11 (**a**)) and in RKO compared to FP (p < 0.001; Figure S11(**a**)). In detailed analysis significantly higher MCP-1 productive capacities were found in RKO compared to FP: at 12 h in the TT (p = 0.001; Figure S11(**c**)) cohort, at 24 h in the SH (p = 0.014; Figure S11(**d**)) and the Fx (p = 0.036; Figure S11(**d**)) cohort. In detailed analysis significantly lower MCP-1 productive capacities were found in FP compared to WT at 12 h in the TT (p = 0.004; Figure S11(**d**)) cohort.

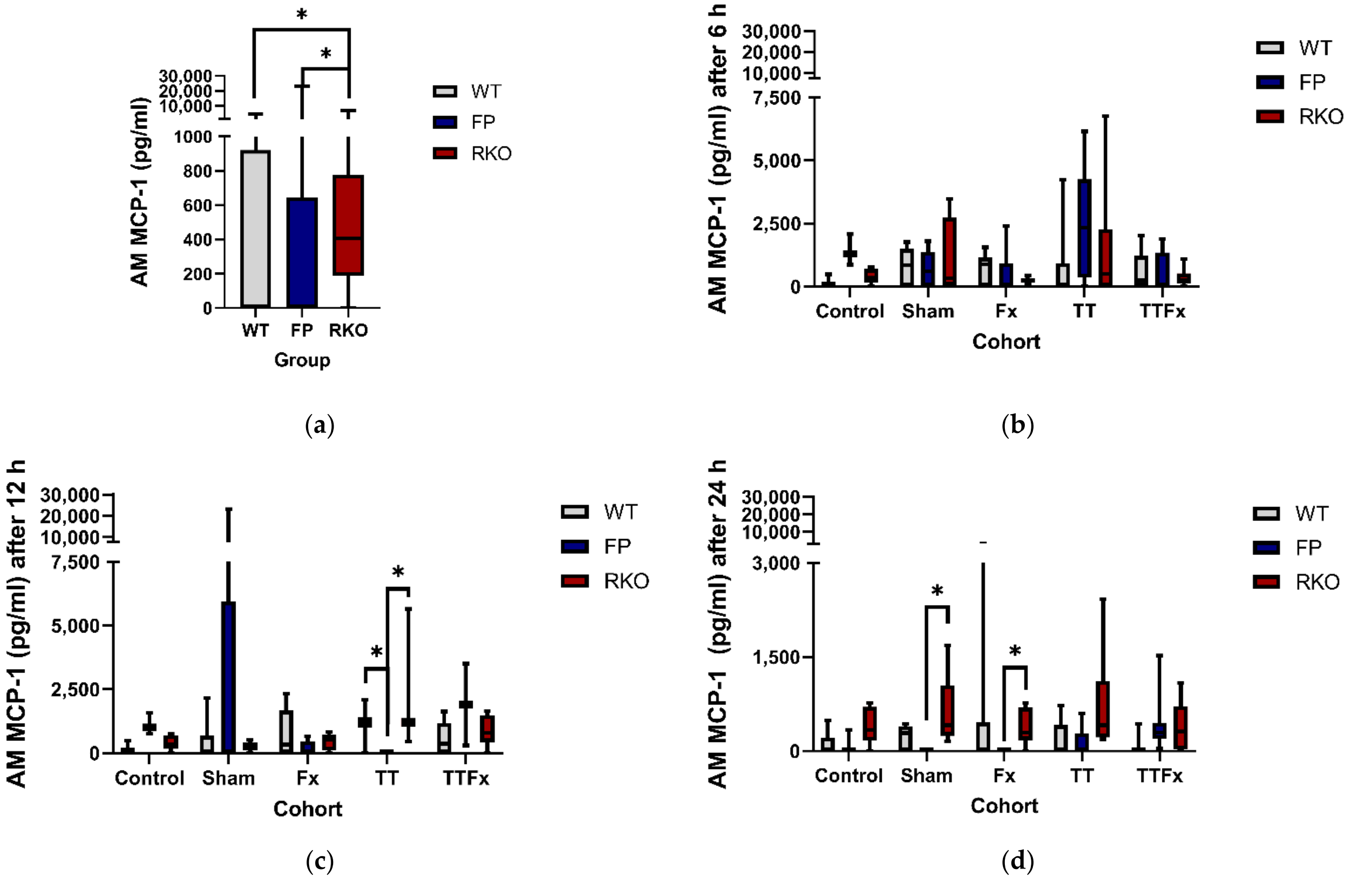

Figure S11. The influence of trauma and IL-6 signaling properties on the posttraumatic MCP-1 productive capacities of alveolar macrophages (AM). Trauma did not significantly influence the MCP-1 productive capacity. Including all cohorts and groups, a significantly higher MCP-1 productive capacity was found in IL-6 receptor animals compared to untreated wildtype animals (WT) and animals treated with sgp130Fc (FP). The MCP-1 productive capacity was significantly higher in RKO compared to FP and in WT compared to FP. Control: healthy animals without trauma generating surgery; Sham: Femur pin stabilization; Fx: femoral fracture; TT: bilateral chest trauma, TTFx: bilateral chest trauma plus femoral fracture. p < 0.05 * *vs.* indicated or % *vs.* Control. (**a**) The posttraumatic MCP-1 productive capacity of AMs, compared by the different IL-6 signaling properties. All animals and time points included. (**b**–**d**) Detailed analysis comparing IL-6 productive capacities of AMs in the different IL-6 signaling properties at different time points after the different interventions.

1.4.4. RANTES

Trauma did not affect RANTES productive capacities, except in one case: We found a significantly lower RANTES productive capacity after trauma in the FP group at 12 h comparing the TT cohort to the CNT cohort (p = 0.046; Figure S12(**c**)) cohort. Including all cohorts and groups, a significantly higher RANTES productive capacity was found in

RKO compared to WT (p = 0.030; Figure S12(**a**)) and FP (p < 0.001; Figure S12(**a**)). In detailed analysis a significantly higher RANTES productive capacity was found in RKO compared to FP at 12 h in the TT (p = 0.021; Figure S12(**c**)) cohort.

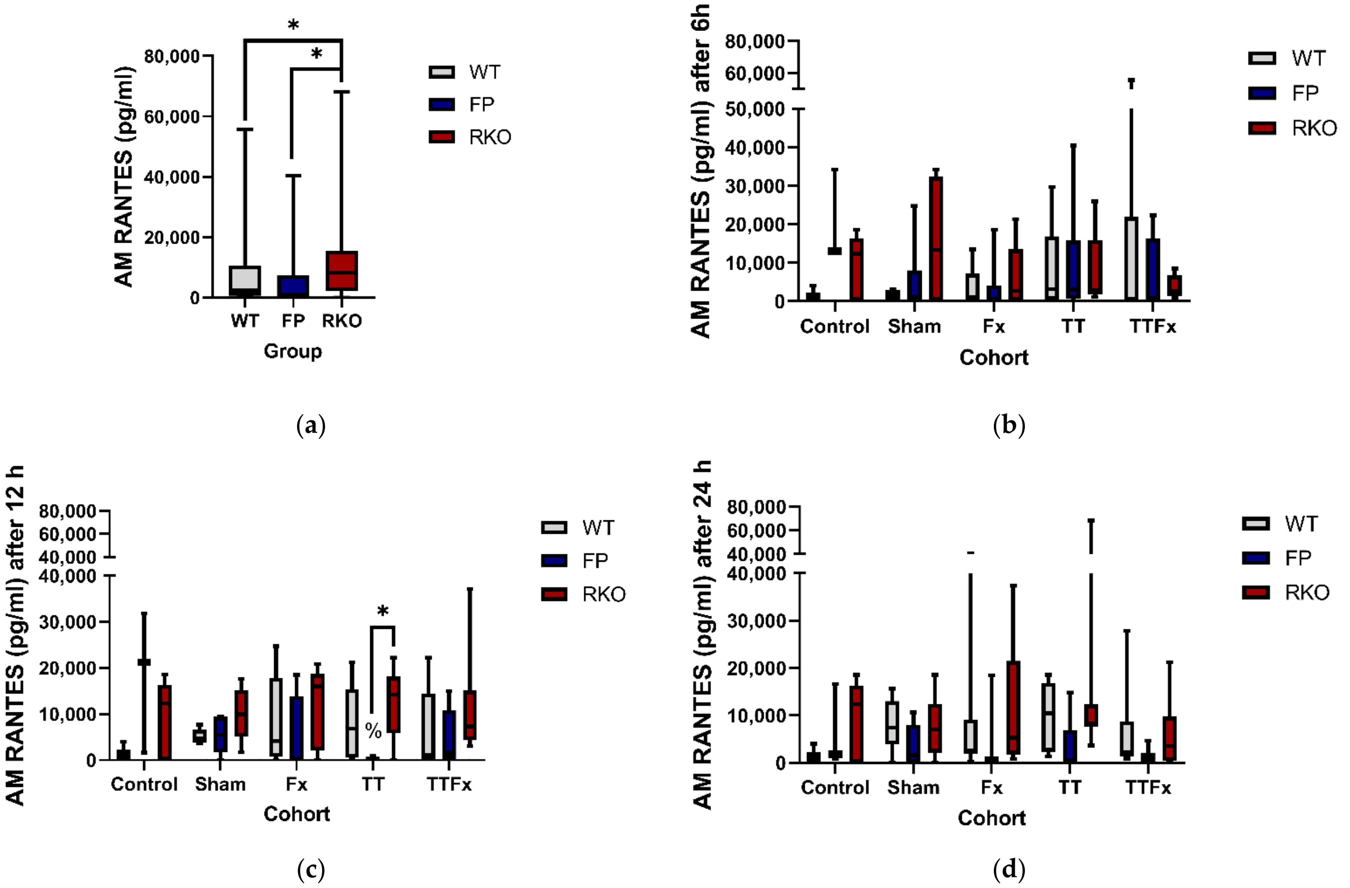

Figure S12. The influence of trauma and IL-6 signaling properties on the posttraumatic RANTES productive capacities of alveolar macrophages (AM). Trauma affected the RANTES productive capacities, only in one case. Including all cohorts and groups, a significantly higher RANTES productive capacity was found in receptor knockout animals (RKO) compared to untreated wildtype animals (WT) and animals treated with sgp130Fc (FP). Control: healthy animals without trauma generating surgery; Sham: Femur pin stabilization; Fx: femoral fracture; TT: bilateral chest trauma, TTFx: bilateral chest trauma plus femoral fracture. p < 0.05 * *vs.* indicated or % *vs.* Control. (**a**) The posttraumatic RANTES productive capacity of AMs, compared by the different IL-6 signaling properties. All animals and time points included. (**b**–**d**) Detailed analysis comparing RANTES productive capacities of AMs in the different IL-6 signaling properties at different time points after the different interventions.

1.4.5. GM-CSF

Trauma did not affect GM-CSF productive capacities. We found a significantly lower GM-CSF productive capacity in FP compared to RKO at 24 h in the TT (p = 0.009; Figure S13(**d**)) cohort. GM-CSF productive capacities were otherwise unaffected by IL-6 signaling.

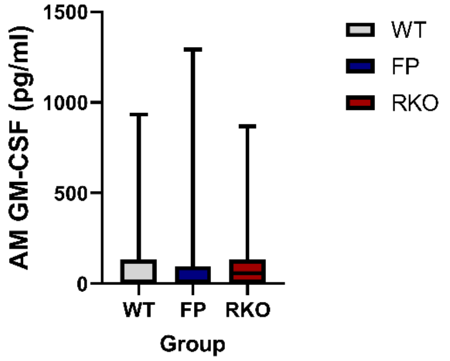

(**a**)

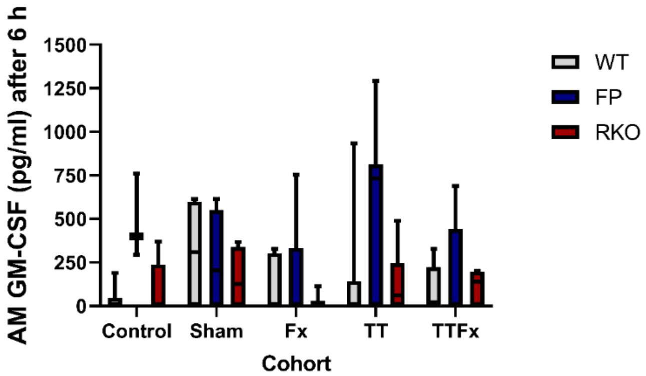

(**b**)

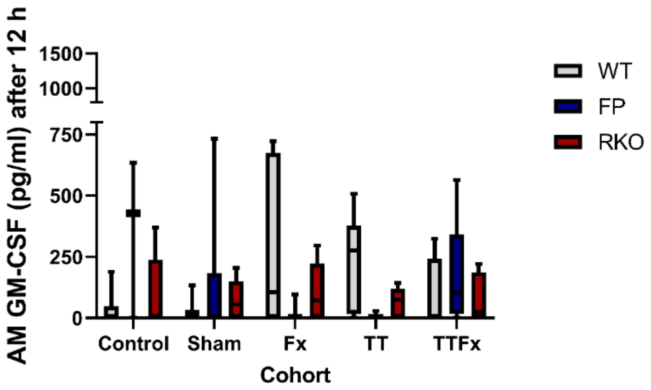

(**c**)

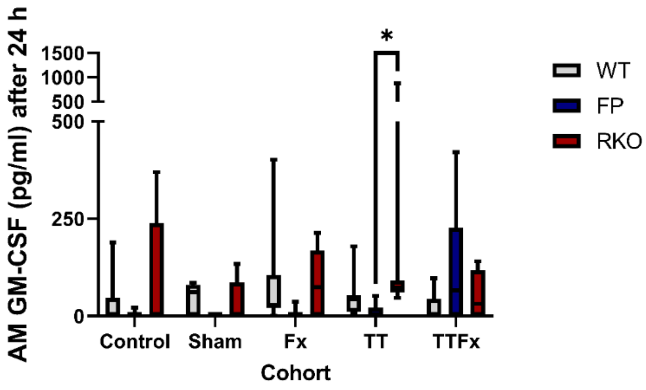

(**d**)

Figure S13 The influence of trauma and IL-6 signaling properties on the posttraumatic GM-CSF productive capacities of alveolar macrophages (AM). Trauma did not affect GM-CSF productive capacities in AMs. The GM-CSF productive capacity was significantly in animals treated with sgp130Fc (FP), compared to receptor knockout animals (RKO) at 24 h in the TT cohort but otherwise unaffected IL-6 signaling properties. There were no significant differences compared to untreated wildtype animals (WT). Control: healthy animals without trauma generating surgery; Sham: Femur pin stabilization; Fx: femoral fracture; TT: bilateral chest trauma, TTFx: bilateral chest trauma plus femoral fracture. $p < 0.05$ * *vs.* indicated or % *vs.* Control. (**a**) The posttraumatic GM-CSF productive capacity of AMs, compared by the different IL-6 signaling properties. All animals and time points included. (**b**–**d**) Detailed analysis comparing GM-CSF productive capacities of AMs in the different IL-6 signaling properties at different time points after the different interventions.

1.4.6. MIP-1β

Trauma causes a significantly higher MIP-1β productive capacity in one case. In WT at 24 h MIP-1β was measured significantly higher in TT compared to CNT ($p = 0.004$; Figure S14(**d**)). Including all cohorts and groups, a significantly lower MIP-1β productive capacity was found in FP compared to WT ($p = 0.002$; Figure S14(**a**)) and FP compared to RKO ($p < 0.001$; Figure S14 (**a**). In detailed analysis significantly lower MIP-1β productive capacities were found in FP compared to RKO: at 12 h in the Fx ($p = 0.011$; Figure S14(**c**)) cohort and in the TT ($p < 0.001$ Figure S14 (**c**)) cohort, at 24 h in the Fx ($p = 0.028$; Figure S14(**d**)) cohort. In detailed analysis we found significantly lower MIP-1β productive capacity in FP compared to WT: at 24 h in the TT cohort ($p = 0.003$; Figure S14(**d**)). There were no significant differences comparing RKO and WT in the different trauma cohorts at different times.

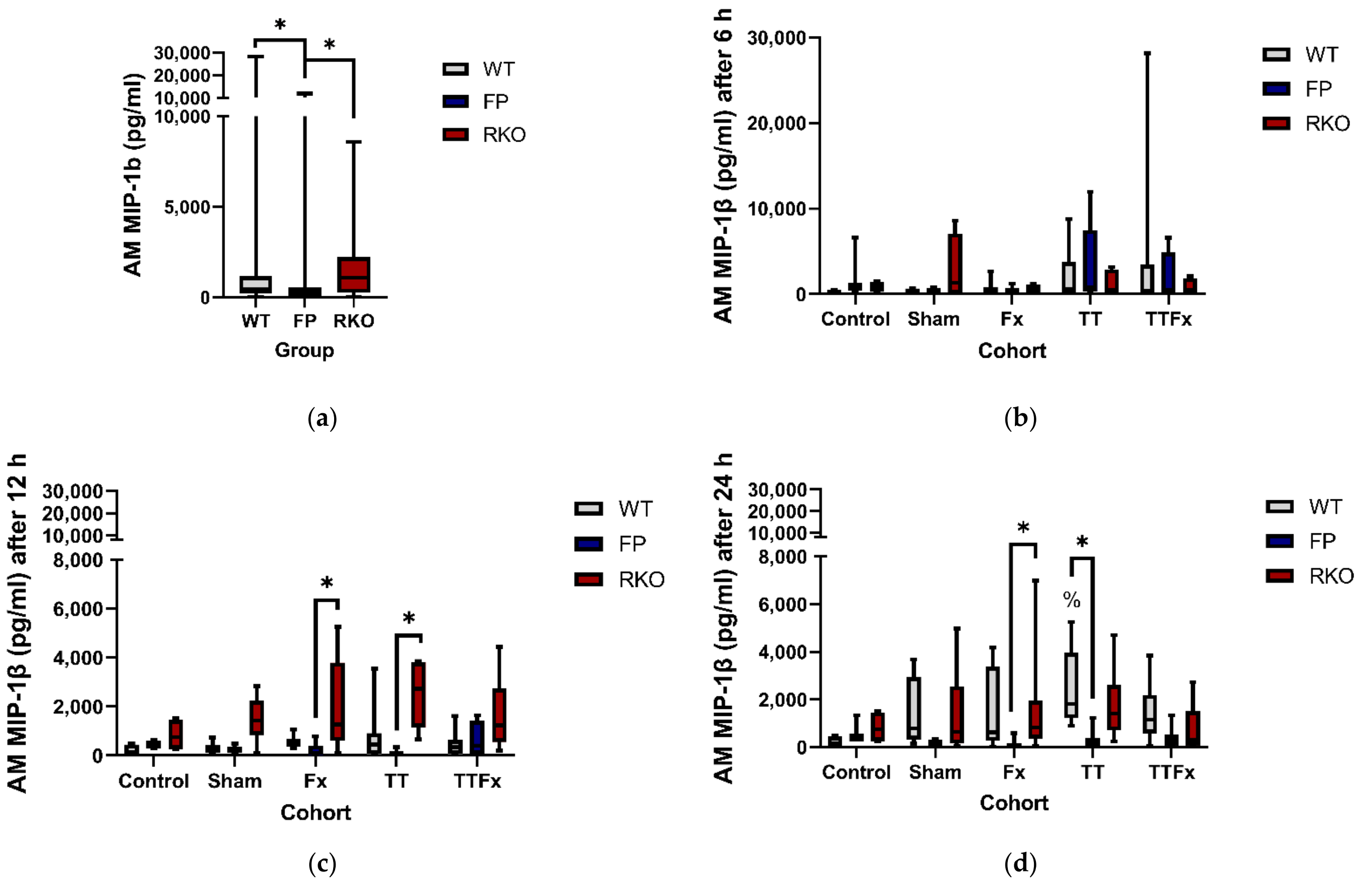

Figure S14. The influence of trauma and IL-6 signaling properties on the posttraumatic MIP-1β productive capacities of alveolar macrophages (AM). Trauma causes a significantly higher MIP-1β productive capacity untreated wildtype animals (WT) in the TT cohort at 24 h. The MIP-1β productive capacity was lower in animals treated with sgp130Fc (FP) compared to IL-6 receptor knockout animals (RKO) and untreated wildtype animals (WT). Control: healthy animals without trauma generating surgery; Sham: Femur pin stabilization; Fx: femoral fracture; TT: bilateral chest trauma, TTFx: bilateral chest trauma plus femoral fracture. $p < 0.05$ * *vs.* indicated or % *vs.* Control. (**a**) The posttraumatic MIP-1β productive capacity of AMs, compared by the different IL-6 signaling properties. All animals and time points included. (**b**–**d**) Detailed analysis comparing MIP-1β productive capacities of AMs in the different IL-6 signaling properties at different time points after the different interventions.
